# Transfer of inflammatory mitochondria via extracellular vesicles from M1 macrophages induces ferroptosis of pancreatic beta cells in acute pancreatitis

**DOI:** 10.1002/jev2.12410

**Published:** 2024-02-06

**Authors:** Yuhua Gao, Ningning Mi, Wenxiang Wu, Yuxuan Zhao, Fangzhou Fan, Wangwei Liao, Yongliang Ming, Weijun Guan, Chunyu Bai

**Affiliations:** ^1^ Precision Medicine Laboratory for Chronic Non‐communicable Diseases of Shandong Province, Institute of Precision Medicine Jining Medical University Jining Shandong China; ^2^ Institute of Animal Sciences Chinese Academy of Agricultural Sciences Beijing China; ^3^ College of Animal Science and Technology, College of Veterinary Medicine Zhejiang A&F University Lin'an China

**Keywords:** extracellular vesicles, ferroptosis, M1 macrophages, mitochondria, pancreatic beta cells

## Abstract

Extracellular vesicles (EVs) exert a significant influence not only on the pathogenesis of diseases but also on their therapeutic interventions, contingent upon the variances observed in their originating cells. Mitochondria can be transported between cells via EVs to promote pathological changes. In this study, we found that EVs derived from M1 macrophages (M1‐EVs), which encapsulate inflammatory mitochondria, can penetrate pancreatic beta cells. Inflammatory mitochondria fuse with the mitochondria of pancreatic beta cells, resulting in lipid peroxidation and mitochondrial disruption. Furthermore, fragments of mitochondrial DNA (mtDNA) are released into the cytosol, activating the STING pathway and ultimately inducing apoptosis. The potential of adipose‐derived stem cell (ADSC)‐released EVs in suppressing M1 macrophage reactions shows promise. Subsequently, ADSC‐EVs were utilized and modified with an F4/80 antibody to specifically target macrophages, aiming to treat ferroptosis of pancreatic beta cells in vivo. In summary, our data further demonstrate that EVs secreted from M1 phenotype macrophages play major roles in beta cell ferroptosis, and the modified ADSC‐EVs exhibit considerable potential for development as a vehicle for targeted delivery to macrophages.

## 1 INTRODUCTION

Acute pancreatitis (AP) is an inflammatory disease of the pancreas (Norberg et al., 2018) that causes post‐pancreatitis diabetes mellitus (PPDM) (Pendharkar et al., 2017; Tu et al., 2017). During the onset of AP, an acute accumulation of macrophages is observed in the pancreas. These cells are among the first cell types to infiltrate the islets and continuously secrete inflammatory factors (Wu et al., 2020). Macrophages are detrimental to the function and survival of pancreatic beta cells and contribute to beta cell failure in both type 1 and 2 diabetes mellitus (Van Gassen et al., 2015). Intercellular mitochondrial transfer is a novel cell‐to‐cell communication mechanism that involves various pathways, such as tunnelling nanotubes (TNTs), extracellular vesicles (EVs), and naked mitochondrial extrusion (J. Chen et al., 2021; H. Y. Z. Li et al., 2019). Mitochondrial transfer by macrophages plays a significant role in the pathology of heart failure (J. Chen, Fu et al., 2022; Y. H. Liu, Wu et al., 2022; Nicolás‐Avila et al., 2020), inflammatory pain (van der Vlist et al., 2022), and osteoporosis (Cai et al., 2023). However, the specific mechanisms and functions of mitochondrial transfer during beta‐cell failure remain unclear.

EVs are nanometre‐scale vesicles (50–200 nm in diameter) that exhibit “dish” shapes and are released by numerous cell types, including immunocytes and pancreatic islets (Guay et al., 2019; Z. Liu et al., 2018; Santulli, 2018; Saravanan et al., 2019). EVs are secreted from donor cells and transport nucleic acids and proteins that can be transferred in an active form into recipient cells to influence cellular functions, such as proliferation and apoptosis. Pancreatic beta cells undergo failure and apoptosis upon receiving EVs from CD4^+^ and CD8^+^ T lymphocytes (Guay et al., 2019). Macrophages are also involved in pancreatitis (Wu et al., 2020). M1 macrophages dominate the proinflammatory phase in AP mice, whereas M2 macrophages participate in pancreatic repair. EVs derived from M1 macrophages play a critical role in the inflammatory response (Xie et al., 2022; Yuan et al., 2017). However, there is a lack of clarity regarding mitochondrial transfer from M1 macrophages to beta cells through EVs and their potential role in inducing beta cell failure and apoptosis during the proinflammatory phase of AP.

Mesenchymal stem cell (MSC)‐secreted EVs reverse the macrophage phenotype from M1 to M2, and are used to treat rheumatoid arthritis and spinal cord injury. Moreover, melatonin pretreatment promotes the reversal of EVs on reversing the M1 macrophage phenotype (C. Liu, Hu et al., 2022; You et al., 2021). However, the effects of MSC‐derived EVs on reversing macrophage polarization in the pancreas to treat abnormal glucose metabolism in AP remain unknown. In this study, pancreas‐resident macrophages and AP mice were used to investigate the role of proinflammatory macrophages in beta cell failure and apoptosis, the potential of EVs derived from adipose‐derived stem cells (ADSCs‐EVs) in ameliorating macrophage polarization, and the improvement of functional recovery of pancreatic beta cells in vitro and in vivo.

## 2 MATERIALS AND METHODS

### 2.1 Isolation and culture of pancreas‐resident macrophages

Pancreas‐resident macrophages were isolated using a previously described procedure for mouse tissue macrophage isolation (Alonso‐Herranz et al., 2019) with some modifications. Briefly, pancreatic tissue was cut into small pieces (approximately 1 mm^3^) and digested using collagenase P (Roche, Switzerland) to obtain a single‐cell suspension. Antibodies against CD45, CD11b, (Cell Signalling Technology, Danvers, MA, 98819, 35476, 1:100) MHC11, F4/80 (Abcam, ab237959, ab270798, 1:100), and magnetic bead‐conjugated anti‐mouse/rabbit IgG (Thermo Fisher) were used to sort macrophages from a single‐cell suspension of pancreatic tissue through magnetic cell separation. CD45‐positive cells were isolated from a single‐cell suspension and cultured in primary macrophage culture medium (SAIOS, PM‐011, China). After 72 h of culture and 80% confluence, the suspended cells and supernatant were discarded, and adherent cells were harvested using 10 mM EDTA. Subsequently, CD11b‐positive cells were selected from a population of CD45‐positive cells. CD11b‐positive cells were sequentially cultured in primary macrophage culture medium and harvested using 10 mM EDTA. CD11b‐positive cells were subsequently used to isolate MHC11‐positive cells. MHC11‐positive cells were cultured in a primary macrophage culture medium until they reached 80% confluence. Finally, F4/80‐positive cells were selected from a subset of MHC11‐positive cells and used for further experiments. Flow cytometry was used to analyze and validate each stage of cell sorting. The resulting positive cells were identified as macrophages. The obtained macrophages were cultured in primary macrophage culture medium. For M1 polarization of macrophages, lipopolysaccharide (LPS; 1 μg/mL, sigma, L4391) and inerferon‐γ (20 ng/mL, Peprotech, 315‐05) were added in cell cultures for 48 h. For M2 polarization of macrophages, IL‐4 (40 ng/mL, Peprotech, 214‐14) was added to cell cultures for 48 h. To isolate EVs, FBS was replaced with knockout serum replacement.

### 2.2 Beta‐TC‐6 cell culture and glucose tolerance test

Mouse Beta‐TC‐6 cells (beta cells) were kindly provided by the Stem Cell Bank, Chinese Academy of Sciences, and cultured in H‐DMEM supplemented with 10% (v/v) FBS, 100 mg/mL streptomycin, and 100 U/mL penicillin after STR authentication. When TC‐6 cells reached 70%−80% confluence, they were subcultured at a 1:3 ratio using 0.25% (m/v) trypsin with 0.01% (m/v) EDTA. To investigate the impact of M1 macrophages on beta cells, a 24‐well Transwell chamber (0.4 μm, BIOFIL, China) was utilized for co‐culturing. The Transwell inserts were seeded with M1 macrophages at a density of 1 ×10^5^ cells per well and pretreated with either 10 μM GW4869 or an equivalent volume of dimethyl sulfoxide (DMSO) as a vehicle for 24 h according to previous reports (Ding et al., 2021; Wang et al., 2023). Additionally, Beta‐TC‐6 cells (1 ×10^5^ cells/well) were seeded in 24‐well cell culture plates and cultured for 24 h. Transwell inserts, which were inoculated with M1 macrophages, were washed thrice with phosphate‐buffered saline (PBS), 5 min/wash, and then inserted and suspended above Beta‐TC‐6 cells and co‐cultured in H‐DMEM supplemented with 10% (v/v) FBS, 100 mg/mL streptomycin, and 100 U/mL penicillin for 24 h. To determine whether insulin release from TC‐6 cells was dependent on glucose, we used two glucose concentrations (2 and 20 mM) and measured insulin release as previously described (Pagliuca et al., 2014). In summary, the cells were washed twice with Krebs buffer (Solarbio Bio, G0430, China), followed by a pre‐incubation period of 2 h in 2 mM glucose Krebs buffer to eliminate any remaining insulin. Subsequently, Beta TC‐6 cells were washed twice with Krebs buffer, incubated in 20 mM glucose‐Krebs buffer for 30 min, and the supernatant was collected. The supernatant was used to quantify the insulin levels using an enzyme‐linked immunosorbent assay (ELISA) kit (Sinogenes Biotech, China). ELISA was conducted in accordance with the manufacturer's instructions and the plate was scanned using a microplate reader (Bio‐Rad 680).

### 2.3 EVs isolation from macrophages and their characterization

To obtain EVs derived from macrophages, fetal bovine serum (FBS, PAN Biotech, P30‐3302, Germany) devoid of EVs was used for macrophage culture. The EVs were subsequently isolated from the culture supernatant via ultracentrifugation. Initially, the freshly obtained culture supernatant was subjected to centrifugation at 800 *g* for 5 min at 4°C to eliminate macrophages, followed by centrifugation at 2,000 *g* for 10 min and 10,000 *g* for 30 min at 4°C to remove cellular debris. Subsequently, the supernatant was subjected to ultracentrifugation at 100,000 *g* for 2 h at 4°C using a Beckman Coulter Optima XPN‐100 Ultracentrifuge equipped with an SW 41 Ti rotor. The supernatant was discarded, and the pellet was washed using 12 mL of PBS. This was followed by a second ultracentrifugation at 100,000 *g* for 2 h at 4°C. The resulting supernatant was discarded, and the EVs were resuspended in 100 μL of PBS. Purified EVs were characterized by electron microscopy, NTA, and immunoblot analyses as previously described (J. Li et al., 2013). To investigate the potential role of purified EVs in beta cell failure and apoptosis, Beta TC‐6 cells were incubated with various concentrations of the EVs for 48 h at 37°C.

### 2.4 Transmission electron microscopy

A 10 μL volume of EVs with a concentration of 1×10^8^ particles/mL was applied onto a copper grid for a precipitation period of 1 min, followed by removal of the supernatant. Subsequently, 10 μL of uranyl acetate solution was applied onto the copper grid for a precipitation period of 1 min, followed by removal of the supernatant. The samples were then air‐dried at room temperature for 10 min and examined using transmission electron microscopy (JEM1400, JEOL, Japan) at an acceleration voltage of 100 kV.

### 2.5 Nanoparticle tracking analysis (NTA)

The particle size and concentration of EVs were assessed using NTA using a Zetaview‐PMX120‐Z instrument (Particle Metrix, Meerbusch, Germany) and the corresponding software, ZetaView (version 8.05.14 SP7). To measure particle size and concentration, the isolated EV samples were diluted 1:100 in 1× PBS buffer. NTA measurements were conducted at 11 positions, and the ZetaView system was calibrated using 110 nm polystyrene particles. The temperature was maintained at approximately 23−30°C.

### 2.6 Immune electron microscopy

First, 100 μL of EVs (1×10^8^ particles/mL) was combined with 4% paraformaldehyde (PFA) in a 1:1 ratio, and 20 μL of the mixture was deposited onto a pristine plastic film. The front side of the carbon grid used for electron microscopy was positioned on the droplet and allowed to remain undisturbed for 20 min. Next, 100 μL of PBS was introduced, and two 3‐min rinses were performed. Subsequently, 100 μL of a 50 mM glycine solution was added, and three 3‐minute rinses were carried out. Following this, 100 μL of a 5% bovine serum albumin (BSA) solution was added, followed by incubation for 10 min. Then, 20 μL of the ATP5A primary antibody working solution was added and the sample was incubated at room temperature for 3 h. The carbon grid was transferred to 100 μL of wash buffer, and six 3‐min rinses were conducted. Next, 20 μL of a 1:100 diluted colloidal gold secondary antibody was added followed by incubation at room temperature for 1 h. Subsequently, the sample was rinsed six times for 3 min each time using PBS containing 0.5% BSA. Following this, the sample was rinsed six times for 2 min each time using 100 μL of PBS. A 1% glutaraldehyde solution (100 μL) was added and the sample was incubated at room temperature for 2 min. The sample was then rinsed six times for 2 min each time using 100 μL of deionized water. Subsequently, 10 μL of phosphotungstic acid negative stain was applied for 90 s, and the sample was allowed to air dry. Afterwards, machine detection was performed using transmission electron microscopy (JEM1400, JEOL, Japan).

### 2.7 Mitochondria isolation and transfer into beta‐TC‐6 cells

The CellLight® Mitochondria‐GFP (Thermo Fisher C10600), a fusion construct comprising the E1 α‐ketoglutarate dehydrogenase leader sequence and Tag GFP, was introduced into macrophages at a concentration of 2 μL per 10^4^ cells for overnight incubation, serving the purpose of labelling mitochondria. GFP‐labelled mitochondria were isolated from the culture supernatant of macrophages using a previously reported method (J. Chen, Fu et al., 2022). The freshly obtained culture supernatant was subjected to differential centrifugation, starting with centrifugation at 800 *g* for 5 min at 4°C. The resulting supernatant was transferred to a new centrifuge tube and centrifuged at 2,000 *g* for 10 min. The supernatant was then subjected to centrifugation at 10,000 *g* for 30 min at 4°C. The mitochondria were collected from the bottom of the centrifuge tube and subsequently characterized using electron microscopy and flow cytometry (FCM). The mitochondria of Beta‐TC‐6 cells were labelled using MitoTracker Deep Red (Beyotime Biotech, C1034, China), whereas the mitochondria of macrophages were labelled using GFP. The Beta‐TC‐6 cells were seeded in a 24‐well cell culture plate and co‐cultured with macrophages for 48 h to observe the presence of red fluorescence in the Beta‐TC‐6 cells. Mitochondria were quantified using the Bradford Protein Assay Kit (Beyotime Biotechnology, P0006, China). Subsequently, the isolated mitochondria were incubated with Beta TC‐6 cells for 48 h to observe the presence of green fluorescence. Nuclei were labelled with Hoechst 33342 (Beyotime Biotechnology, C1002, China).

### 2.8 Whole‐mtDNA amplification in EVs derived from macrophages

Total DNA from EVs was isolated using a Fast Pure Cell DNA Isolation Mini Kit (Vazyme, DC112, China) following the manufacturer's instructions. A suspension of 1×10^6^ particles/mL of EVs was prepared by resuspending them in 220 μL of PBS. Subsequently, 10 μL of RNase solution and 20 μL of proteinase K were added to the EVs suspension, which was then allowed to stand at room temperature for 15 min. Following this, 250 μL of buffer GB was added to the EVs sample, which was then subjected to a water bath at 65°C for 30 min after thorough vortexing and mixing. The resulting mixture was combined with 180 μL of absolute ethyl alcohol and thoroughly mixed. The mixture was then added to an adsorption column and centrifuged at 12,000 rpm for 1 min. To remove any residual proteins, 500 μL of washing buffer A was added to the adsorption column, which was then centrifuged at 12,000 rpm for 1 min. To remove residual ions, 650 μL of washing buffer B was added to the adsorption column and centrifuged at 12000 rpm for 1 min. This step was repeated twice. Next, the adsorption column was centrifuged at 12000 rpm twice for 2 min each to remove residual ethanol. The adsorption column was then placed in a new 1.5 mL centrifuge tube. Next, 60 μL of Elution Buffer preheated to 70°C was added to the centre of the membrane of the adsorption column. The mixture was incubated at room temperature for 3 min and centrifuged at 12,000 rpm for 1 min. Subsequently, the DNA was then centrifuged in a collection tube. The DNA concentration was measured using a NanoDrop 2000c spectrophotometer (Thermo Fisher Scientific). Subsequently, we utilized 5 ng of this DNA for mtDNA amplification using 2×Taq Plus Master Mix (Vazyme, P213, China) and specific primers (listed in Table S1). The cycling conditions for the amplification consisted of an initial 3‐min denaturation step at 95°C, followed by 30 cycles of 30 s at 95°C, 30 s at 60°C, 60 s at 72°C, and a final extension step of 5 min at 72°C. The resulting PCR products were visualized using 1% agarose gel electrophoresis and subjected to Sanger sequencing. The obtained sequences were functionally annotated using a mitochondrial map. A mitochondrial map was created using the Tutools platform (https://www.cloudtutu.com), which is a free online data analysis website.

### 2.9 Detection of mitochondrial membrane potential in beta TC‐6 cells

The mitochondrial membrane potential of Beta TC‐6 cells was assessed using the JC‐1 Assay Kit according to the manufacturer's instructions (Beyotime Biotechnology, China). In brief, Beta TC‐6 cells were harvested after different treatments and subjected to a 20‐min incubation with JC‐1 dye at 37°C. Following a wash step with JC‐1 staining buffer, the JC‐1 fluorescence of Beta TC‐6 cells was quantified using flow cytometry (CytoFLEX, Beckman Coulter, China). A lower ratio of JC‐1 aggregates to monomers signifies a reduction in mitochondrial membrane potential.

### 2.10 Analysis of apoptosis in beta TC‐6 cells

Apoptosis of TC‐6 cells was assessed using the Annexin V‐FITC/PI staining method, employing the Annexin V‐FITC cell apoptosis detection kit (Beyotime Biotechnology, C1062, China) according to the manufacturer's guidelines. The cell culture medium was extracted using a centrifuge tube. Subsequently, the cells were washed once with PBS by introducing them into the tube and gently tapping the tube to dislodge cells that adhered to the walls. Cells were enzymatically digested with 0.25% trypsin at ambient temperature until they shrank and assumed a spherical shape. Subsequently, trypsin was removed, and the collected cell culture medium was introduced, employing gentle pipetting motion to homogenize the mixture. The resulting mixture was transferred to a centrifuge tube and centrifuged 1000 *g* for 5 min. The supernatant was discarded, and the cells were collected. The cells were gently resuspended in PBS and counted. A total of 1×10^5^ cells were centrifuged at 1000 *g* for 5 min. The supernatant was discarded, and the cells were gently resuspended in 195 μL of Annexin V‐FITC binding solution. Additionally, 5 μL of Annexin V‐FITC and 10 μL of propidium iodide were added, mixed gently, and incubated at room temperature in the dark for 20 min. Apoptosis was quantified using a flow cytometer (CytoFLEX; Beckman Coulter).

### 2.11 Analysis of ferrous ion (Fe^2+^) concentration and lipid peroxidation content in beta TC‐6 cells

The intracellular Fe^2+^ and mitochondrial lipid peroxidation of TC‐6 cells after different treatments were assessed using a FerroOrange fluorescent probe (DOJINDO, F374, Japan) and MitoPeDPP fluorescent probe (DOJINDO, M466, Japan), respectively, following the manufacturer's instructions. Briefly, Beta TC‐6 cells were seeded in a laser confocal microscope dish, treated with M1‐EVs or M1‐mitochondria for 24 h, and then the supernatant was discarded, and the cells were washed twice with PBS. These cells were incubated with 1 μmol/L FerroOrange or 0.5 μmol/L MitoPeDPP at 37°C for 20 min in a cell incubator. Subsequently, the supernatant was discarded and the cells were washed twice using PBS. The cells were then examined using a confocal fluorescence microscope (LeicaSP8, Germany). The intracellular fluorescence intensity of the probes was quantified using the ImageJ software.

### 2.12 GSH/GSSG ratio assay

The ratio of reduced glutathione (GSH) to oxidized glutathione (GSSG) was measured using a GSSG/GSH Quantification Kit according to the manufacturer's instructions (DOJINDO, G263, Japan). Initially, a total of 10^7^ cells were introduced into 80 μL of a 10 mM hydrochloric acid (HCl) solution, and the cells were subjected to two cycles of freeze‐thawing to induce lysis. Subsequently, 20 μL of a 5% solution of sulfosalicylic acid (SSA) was added, and the resulting mixture was subjected to centrifugation at 8,000 *g* for 10 min. The resulting supernatant was carefully transferred to a fresh tube and diluted with deionized water (ddH_2_O) to achieve a final SSA concentration of 0.5%. To prepare the (GSSG) sample tube, 200 μL of the sample and 20 μL of masking solution were combined in a microtube and thoroughly mixed using a vortex mixer. For the measurement of total glutathione, 200 μL of the sample and 20 μL of ddH_2_O were added to a tube and mixed well using a vortex mixer. Subsequently, 40 μL of either GSSG sample or GSH sample was added to each well, followed by 60 μL of Buffer Solution. The mixture was then incubated at 37°C for 1 h. Following this, 60 μL of substrate working solution and 60 μL of enzyme/coenzyme working solution were added to each well, and the mixture was incubated at 37°C for 10 min. Total glutathione and GSSG levels were determined by measuring absorbance at 405 nm. GSH/GSSG = (total glutathione‐ GSSG)/GSSG.

### 2.13 Mitochondrial respiration detection

The Beta‐TC‐6 cells were pretreated with 1 μM Ferrostatin‐1 (Fer 1, HY‐100579), an inhibitor for ferroptosis obtained from MedChemExpress, for 6 h. This group is subsequently referred to as the Fer 1 group. A total of 5×10^3^ normal Beta‐TC‐6 cells or Fer 1‐treated Beta TC‐6 cells were initially placed in XF24 cell culture microplates (Agilent) and cultured with either 1×10^8^ particles/mL M1‐EVs or 25 μg/mL M1 mitochondria for 24 h. A Seahorse XFe24 Analyzer (Agilent) was used to measure oxygen consumption. Following baseline measurements, injections of 1.5 mM oligomycin, 0.5 mM FCCP, and 0.5 mM rotenone/antimycin A were administered sequentially. These drugs were all included in the Cell Mito Stress Test Kit specifically designed for XFe/XF Analyzers (Agilent Technologies, Santa Clara, CA). All rates were adjusted to account for the cellular protein content, which was determined using the Micro BCA assay. The reported data represent the average value ± standard error of the mean obtained from three separate wells.

### 2.14 Immunofluorescence

Cells were seeded on glass coverslips, washed three times with PBS, fixed for 10 min with 4% PFA at 37°C, and then cells were washed three times with PBS and permeabilized for 1 h in block solution (1% BSA, 22.52 mg/mL glycine and 0.1% Tween 20 in PBS). Primary antibodies (TOM20 Polyclonal antibody, 11802‐1, and TOM20 Monoclonal antibody, 66777‐1, 1:200; Proteintech, China; cGAS (1:200, Affinity, China); dsDNA, 1:60, Merck; IκBα (1:100, Cell Signalling Technology); and NF‐κB1 (1:100, Abcam) were diluted in block solution and incubated with cells overnight at room temperature. The cells were washed three times with PBS and incubated with secondary antibodies (anti‐mouse Alexa 488; anti‐rabbit Alexa 594, Proteintech, China, 1:200) for 1 h in the dark. Finally, the cells were incubated with 1 μg/mL DAPI for 15 min and then washed three times with PBS. Images were acquired using a laser scanning confocal microscope (LeicaSP8, Germany).

### 2.15 Human ADSC culture and isolation of ADSC‐derived EVs

Human ADSCs were cultured in OriCell^®^ medium for human adipose‐derived MSCs (OriCell, 90011, Cyagen, China). ADSCs were authenticated and confirmed to be mycoplasma‐free before use. To analyze the biological characteristics of human ADSCs, the main markers of ADSCs, CD29, CD105, and CD90 (Santa Cruz, sc‐9970, sc‐18838, sc‐53456, 1:100) were detected using flow cytometry. Chondrogenic, osteogenic, and adipocyte differentiation of human ADSCs were performed with specific media (OriCell, 90021, 90031, Cyagen, China). To obtain ADSC‐derived EVs, OriCell^®^ medium was replaced by DMEM/F12 (Servicebio, G4611, China) supplemented with 10% knockout serum replacement (Gibco, 10828), 10 ng/mL bFGF (Perprotech, 100–18), 10 ng/mL EGF (Perprotech, 100–15), and 10 μM melatonin (MedChemExpress, B0075). The culture supernatant was used to isolate EVs by ultracentrifugation, as previously described (Tian et al., 2020). Briefly, a freshly prepared culture supernatant was centrifuged at 800 *g* for 5 min at 4°C to remove ADSCs and then at 2,000 *g* for 10 min and 10,000 *g* for 30 min at 4°C to remove cellular debris. Subsequently, the supernatant was centrifuged at 100,000 *g* for 2 h at 4°C in a Beckman Coulter Optima XPN‐100 Ultracentrifuge using an SW 41 Ti rotor. The supernatant was discarded, and the pellet was washed with 12 mL PBS, followed by a second ultracentrifugation at 100,000 *g* for 2 h at 4°C. The supernatant was discarded, and EVs were resuspended in 100 μL PBS. The biological characteristics of the EVs were analyzed using electron microscopy, NTA, and immunoblotting.

### 2.16 Animal model of AP

Six‐week‐old KM mice were purchased from Jinan Pengyue Experimental Animal Breeding Co. Ltd. (Jinan, China), and approximately 100 mice were used in this study, with less than six mice in each group (half male and female). This study was approved by the Committee on the Ethics of Animal Experiments of Jining Medical University (License ID:2017‐JZ‐003). The AP model was established in accordance with previous reports (Sakaguchi et al., 2006; Wu et al., 2020), with some modifications. Mice were randomized in a block design and intraperitoneally injected with cerulein (a cholecystokinin analog, dosage: 50 mg/g every 1 h; Shanghai Yuanye Bio‐Technology Co., Ltd, HY‐A0190) nine times, with the last injection consisting of cerulein with LPS (10 mg/kg, Sigma, St Louis, MO, L2630) to establish the AP model. To assess the AP model, serum amylase levels were measured using an ELISA kit (Sinogenes Biotech). AP mice with serum amylase ≥ 400 pg/mL were used for subsequent experiments. Fasting blood glucose and insulin levels were measured during the AP process at the 1st, 2nd, 3rd, 4th, 5th, and 6th weeks using a glucometer (SANNUO, SANNUO Biotech Ltd., China) and ELISA kits (Solarbio, Beijing, China). All tissue and serum samples from the animals were collected after euthanasia using anesthetic gases. Subsequently, the homeostasis model assessment (HOMA) was used to evaluate the function of β cell (HOMA‐β), HOMA‐β = 20×fasting insulin/(fasting blood glucose ‐ 3.5 mmol/L).

### 2.17 Tracking of ADSC‐derived EVs in vitro and in vivo

To visualize ADSC‐derived EVs in influencing the polarization of macrophages in vitro, EVs were labelled with 2 μM Dil (1,1′‐dioctadecyl‐3,3,3′,3′‐tetramethylindocarbocyanine perchlorate, Coolaber, Beijing, China, Ex/Em = 549/569 nm) for 1 h at 37°C and then purified with a SuperEV 0.5 purification column (Rengen Bio, EXOSEC0.5‐5, China). Macrophages were incubated with various concentrations of EVs for 48 h. The fluorescence intensity of NF‐κB1 (p50, FITC, Ex/Em = 494/518 nm) and IκBα (Cy5, Ex/Em = 649/670 nm) in the nucleus was assessed using a Leica TCS‐SP8 confocal microscope and ImageJ. The Leica TCS‐SP8 confocal microscope was configured with identical parameters, encompassing gain values (DAPI, 805v; FITC, 658 v; Dil, 780 v; Cy5, 858v) and laser energy (Diode 405, 85.2%; OPSL 488, 78.6%; OPSL 552, 81.3%; Diode 638, 83.4%). These settings were employed for the purpose of fluorescence imaging of macrophages subjected to various EVs.

To track ADSC‐derived EVs in vivo, EVs were labelled with 2 μM DiR (Absin, Shanghai, China) for 1 h at 37°C and then purified with the SuperEV 0.5 purification column. Various amounts of EVs were injected intraperitoneally into mice. At 24 h after the injection, the mice were anesthetized and imaged using the NIR‐II Imaging System H (Series III 900/1700, NIROPTICS, Suzhou, China). To further analyze the location of EVs in the tissues, the tissues were selected, fixed, and sectioned, and the DiR signal (Ex/Em = 748/780 nm) was detected using a Nikon TI2‐E+A1 R confocal microscope.

### 2.18 Decoration of ADSC‐derived EVs by an anti‐F4/80 antibody

To analyze the targeted delivery of ADSC‐derived EVs to macrophages, a rat monoclonal anti‐F4/80 antibody (Abcam, ab6640) was attached to the surface of ADSC‐derived EVs using an EV decorating kit (Rengen Biosciences, China) according to the manufacturer's instructions. First, EVs were coupled with Link A by incubating 1×10^10^ EVs with 5 mM Link A for 1.5 h at room temperature, and the reactant was incubated with 2 μM DiR for 1 h at 37°C. This reactant was purified using the SuperEV 0.5 purification column and called DiR‐EVs+Link A. Subsequently, 0.5 mg anti‐F4/80 antibody was incubated with 5 mM linker B for 1.5 h at room temperature and then purified using the protein purification column and called anti‐F4/80+Link B. Ne, DiR‐EVs+Link A and anti‐F4/80+Link B were linked through a click chemical reaction to form DiR‐EVs+Link A‐Link B+anti‐F4/80 compound (called F4/80‐EVs). Finally, the compound was purified using a SuperEV 0.5 purification column and characterized via electron microscopy, NTA, and immunoblotting. To further analyze the location of the DiR‐EVs+Link A‐Link B+anti‐F4/80 compound in the tissues, pancreatic tissues were selected, fixed, sectioned, and immunostained with an anti‐rat antibody (FITC‐conjugated antibody, Zhongshan Golden Bridge, Beijing, China), rabbit monoclonal anti‐F4/80 antibody (Abcam), and anti‐rabbit antibody (Cy5‐conjugated antibody, Zhongshan Golden Bridge).

### 2.19 Statistical analysis

All experiments were independently performed at least three times and repeated in triplicate. Results are presented as the mean ± standard error of the mean (SEM). Differences were assessed using Student's t‐test (two groups) or one‐way ANOVA (more than two groups) unless noted otherwise. Statistical significance was set at *P* < 0.05. A graphical abstract was drawn using the FigDraw tools (www.figdraw.com).

## 3 RESULTS

### 3.1 M1 macrophages transfer mitochondria to pancreatic beta cells through EV release

**FIGURE 1:**
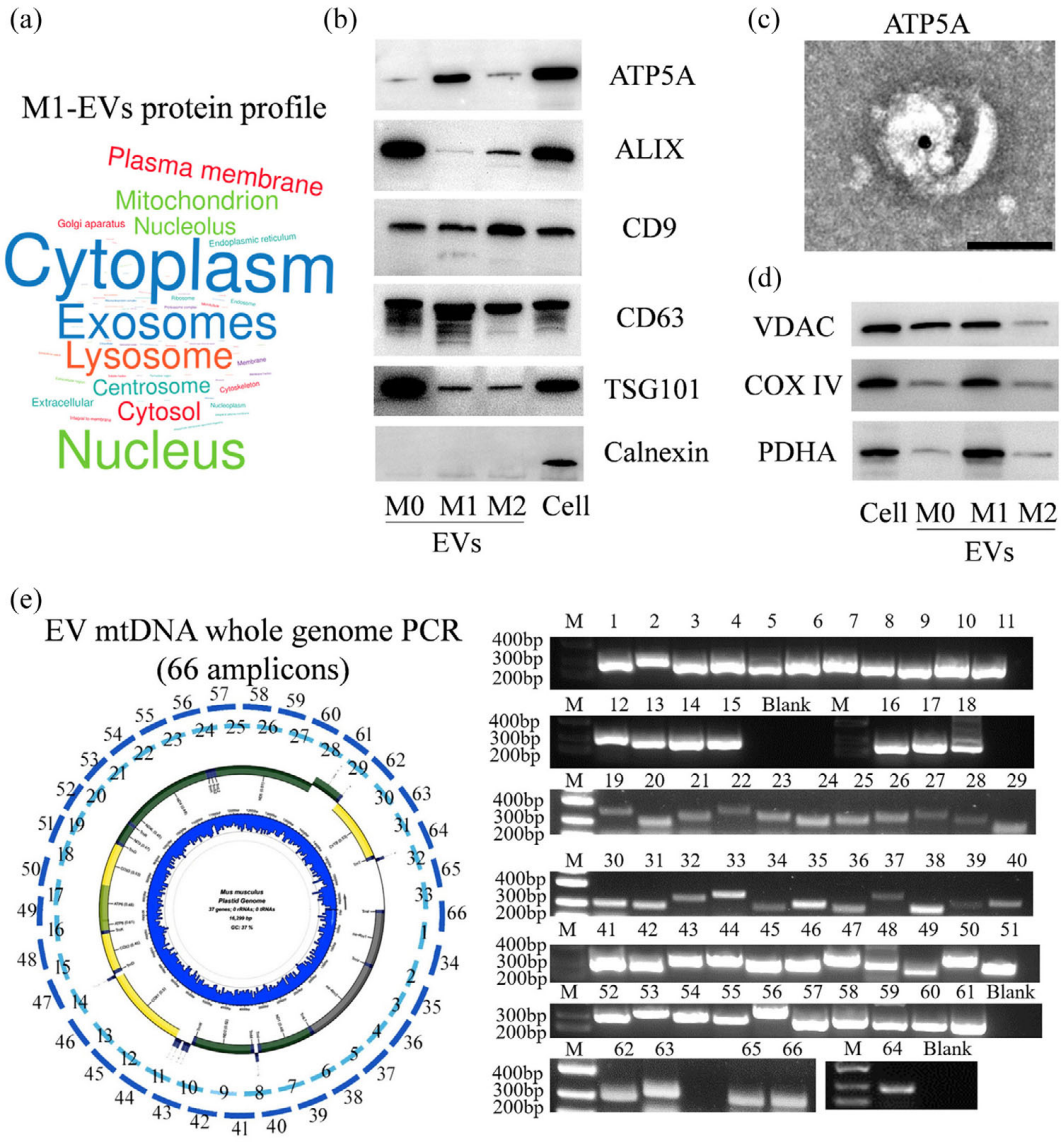
EVs derived from macrophages containing packaged mitochondria. (a) Word cloud image illustrating the subcellular localization annotations found in the differential protein between M1‐EVs and M0‐EVs. The mitochondria component was enclosed within M1‐EVs. The detailed list of differential proteins can be found in Table S1. (b) Western blot analysis was conducted to examine the presence of EV‐positive‐markers, TSG101, CD63, CD9, and Alix, EV‐negative‐markers, Calnexin, as well as the mitochondrial marker ATP5A. (c) Immuno‐electron microscope analysis was performed to investigate the presence of ATP5A in M1‐EVs. (d) Western blot analysis was conducted to assess the presence of mitochondrial major components, including VDAC, COX IV, and PDHA in EVs. (e) Schematic and a representative gel electrophoresis image of whole‐genome amplification (using 66 overlapping PCR amplicons covering the complete mtDNA genome) from M1macrophages‐derived EV‐DNA.

To elucidate the molecular mechanism underlying the apoptotic effect of EVs derived from M1 macrophages (M1‐EVs) on beta cells, pancreatic resident macrophages were first isolated from normal mouse pancreatic tissue (Figure S1), cultured in vitro, and named M0 macrophages. M0 macrophages were then polarized into M1 and M2 phenotypes (Figure S2A–D). Subsequently, the EVs were isolated via ultracentrifugation, and their morphologies and particle sizes are shown in Figure S2E–G. The protein profiles of these EVs were analyzed using proteomic technology and their functional and subcellular localization annotations were classified. The main categories included the cytoplasm, exosomes, lysosomes, nuclei, mitochondria, and plasma membrane (Figure 1a and Table S2). These data suggest that the mitochondrial component is enclosed within EVs prior to their secretion by M1 macrophages. To further validate the transfer of mitochondria via EVs, the presence of ATP5A, a specific mitochondrial marker, was assessed in the EVs via western blotting and immunoelectron microscopy. The findings depicted in Figures 1b,c, and S3 indicate that ATP5A was positively expressed in M1‐EVs. Additionally, the proteins VDAC, COX IV, and PDHA, which are located within the mitochondrial matrix, also demonstrated positive expression in M1‐EVs, as confirmed through western blotting analysis (Figure 1d and Figure S3). These proteins constitute the outer mitochondrial membrane, the inner mitochondrial membrane, and participate in the final stage of the electron transport chain, respectively. This study investigated the presence of mitochondrial components in the EVs secreted by M1 macrophages. However, it remains to be confirmed whether all mitochondria are enclosed within these EVs. To address this, whole‐genome PCR analysis was performed on DNA extracted from M1‐EVs. The results revealed the successful amplification of 66 amplicons corresponding to the entire mitochondrial DNA (mtDNA) genome. Subsequent amplicon sequencing confirmed the encapsulation of complete mitochondria within EVs, which were subsequently transported out of the cells (Figure 1e and Figure S4).

**FIGURE 2:**
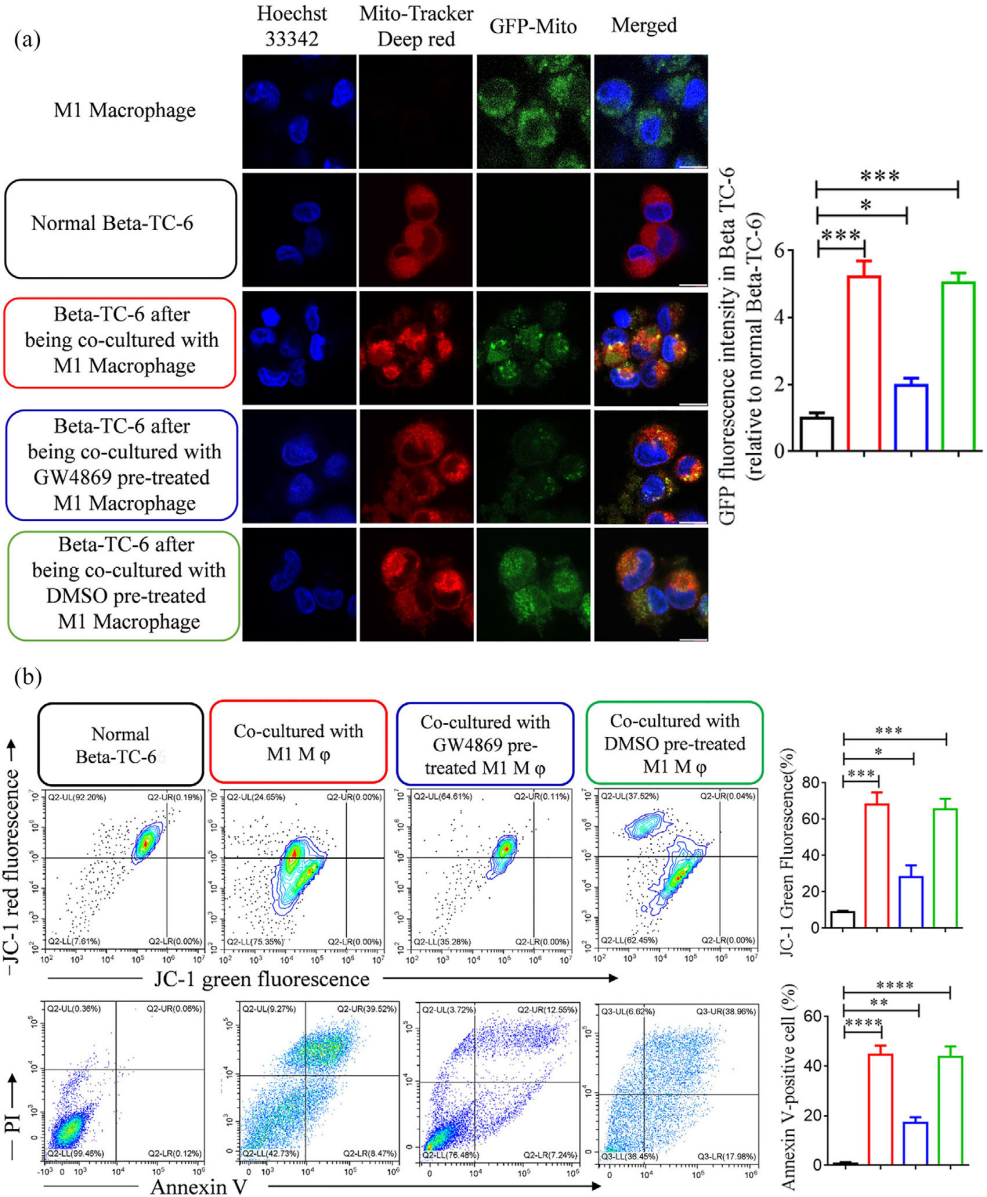
The transfer of mitochondria between M1 macrophages and Beta TC‐6 cells and the subsequent analysis of apoptosis in Beta TC‐6 cells were investigated. (a) Representative images of mitochondria transfer between M1 macrophages and Beta TC‐6 cells (left panel). Additionally, mitochondrial fluorescent quantitation of M1 macrophages in Beta TC‐6 cells was conducted (right panel). (b) Apoptosis analysis was performed in Beta TC‐6 cells following co‐culture with M1 macrophages. This analysis included the examination of mitochondrial membrane potential (upper panel) and the identification of Annexin X‐positive cells (lower panel). *n* = 3, *P < 0.05, **P < 0.01, ***P < 0.001, ****P < 0.0001.

To visually detect the transfer of mitochondria from M1 macrophages to pancreatic beta cells through EVs transport, GFP was used to label the mitochondria in M1 macrophages, whereas MitoTracker Deep Red was used to label the mitochondria in beta cells (Beta TC‐6 cells). Subsequently, the cells were co‐cultured using a Transwell unit, and GFP fluorescence was observed after 48 h. However, the fluorescence intensity was significantly reduced in M1 macrophages pretreated with GW4869 (Figure 2a). GW4869, a well‐established inhibitor of exosome release from macrophages(Essandoh et al., 2015), exhibited significant efficacy in reducing the release of EVs in M1 macrophages, as demonstrated in our study (Figure S5A).

### 3.2 Inflammatory mitochondria derived from M1 macrophages induce apoptosis of beta cells

JC‐1 is a fluorescent probe that is widely used to detect mitochondrial membrane potential. In normal mitochondria, JC‐1 aggregates in the mitochondrial matrix to form polymers and emits strong red fluorescence (Salvioli et al., 1997). When the mitochondrial membrane potential is low, JC‐1 cannot aggregate in the mitochondrial matrix, resulting in a green fluorescence. The mitochondrial membrane potential of TC‐6 cells was assessed using JC‐1 cells following co‐cultivation with M1 macrophages. Our findings revealed a substantial increase in the proportion of positively stained cells exhibiting JC‐1 green fluorescence compared to control cells. However, this ratio was significantly decreased in TC‐6 cells co‐cultured with M1 macrophages pretreated with GW4869 (Figure 2b). In the interim, the apoptosis of Beta TC‐6 was also assessed using the Annexin V‐FITC/PI Apoptosis Detection Kit. These findings were consistent with those obtained using the JC‐1 detection method. Specifically, the proportion of Annexin V‐positive cells in TC‐6 cells co‐cultured with M1 macrophages was substantially higher than that in normal cells. Conversely, a notable decrease in Annexin V‐positive cells was observed in TC‐6 cells co‐cultured with M1 macrophages pretreated with GW4869 (Figure 2b). Our findings suggest that the administration of GW4869 led to a reduction in mitochondrial release from macrophages via extracellular vesicle secretion. Additionally, we observed that treatment with GW4869 resulted in a significant increase in GFP fluorescence in macrophages (Figure S5B). These results suggested that mitochondrial transfer occurred through the release of EVs.

**FIGURE 3:**
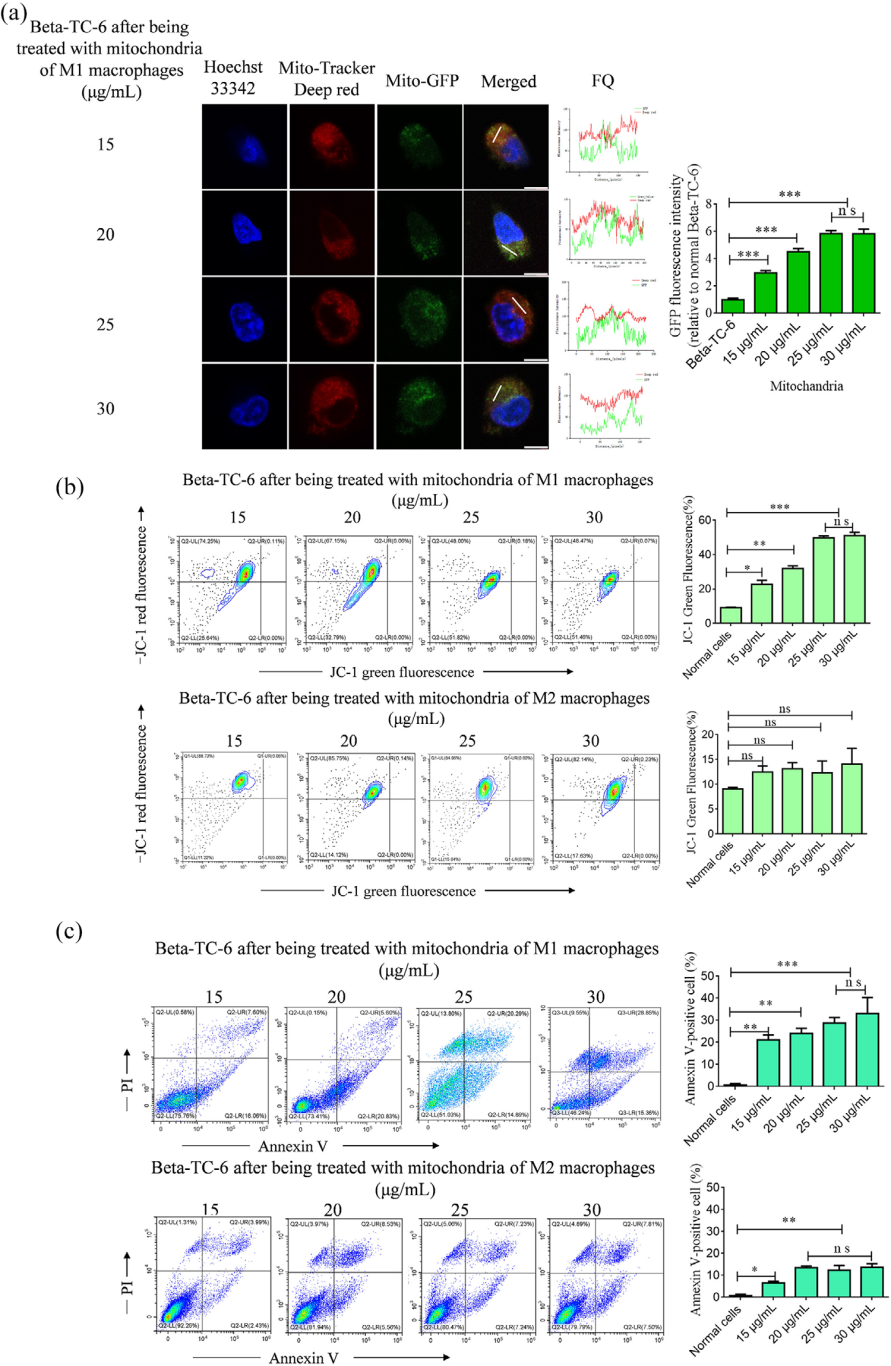
Analysis of apoptosis in Beta TC‐6 cells following incubation with mitochondria derived from macrophages. (a) Representative images demonstrate the incubation of Beta TC‐6 cells with varying concentrations of macrophage mitochondria, green fluorescence indicating macrophage mitochondria and red fluorescence indicating Beta TC‐6 cell mitochondria. Fluorescence quantitation (FQ) was performed to assess the levels of fluorescence. (b) Analysis of mitochondrial membrane potential in Beta TC‐6 cells after treatment with mitochondria derived from M1 or M2 macrophages, with different concentrations being utilized. (c) Analysis of Annexin X‐positive cells in Beta TC‐6 cells following exposure to different concentrations of mitochondria derived from M1 macrophages or M2 macrophages. *n* = 3, *P < 0.05, **P < 0.01, ***P < 0.001. ns, not significant.

Activated macrophages undergo metabolic reprogramming, which induces a proinflammatory phenotype. This metabolic shift is characterized by a transition in macrophage mitochondria from ATP production to the generation of mitochondrial reactive oxygen species (ROS) (Mills et al., 2016). Additionally, this ROS produced by mitochondria are also secreted from macrophages and taken up by other cells, such as myocardial cells, to induce cell apoptosis (J. Chen, Fu et al., 2022). In our study, the mitochondria of M0 macrophages were labelled with GFP, which activated the inflammatory M1 and anti‐inflammatory M2 phenotypes. Subsequently, mitochondria secreted from M1 and M2 macrophages were selected from the cellular supernatant and detected using transmission electron microscopy and flow cytometry, as shown in Figure S6A and B. To visually detect the fusion of mitochondria between pancreatic beta cells and macrophages, mitochondria derived from M1 macrophages labelled with GFP were introduced into Beta TC‐6 cells labelled with MitoTracker Deep Red at varying concentrations. Subsequently, the fluorescence co‐localization was analyzed, and our findings revealed the presence of green fluorescence in Beta TC‐6 cells, with the intensity of green fluorescence progressively increasing as the concentration of mitochondria rose, reaching its peak at 25 and 30 μg/mL. Green fluorescence was co‐localized with the red fluorescence, and the quantification of fluorescence between the red and green signals was observed in the same pixels (Figure 3a). These data provide evidence that the mitochondria of beta cells were fused with mitochondria derived from M1 macrophages. To investigate the effect of inflammatory mitochondria from M1 macrophages on the apoptosis of pancreatic beta cells, we isolated mitochondria from the cellular supernatants of M1 and M2 macrophages and co‐cultured them with Beta TC‐6 cells at varying doses. Our findings suggest a progressive increase in the ratio of apoptotic cells with an increase in mitochondrial concentration in M1 macrophages. This increase reached its peak at concentrations of 25 and 30 μg/mL. However, no significant changes were observed in the mitochondria of M2 macrophages as determined by the detection of JC‐1 and Annexin V (Figure 3b,c).

**FIGURE 4:**
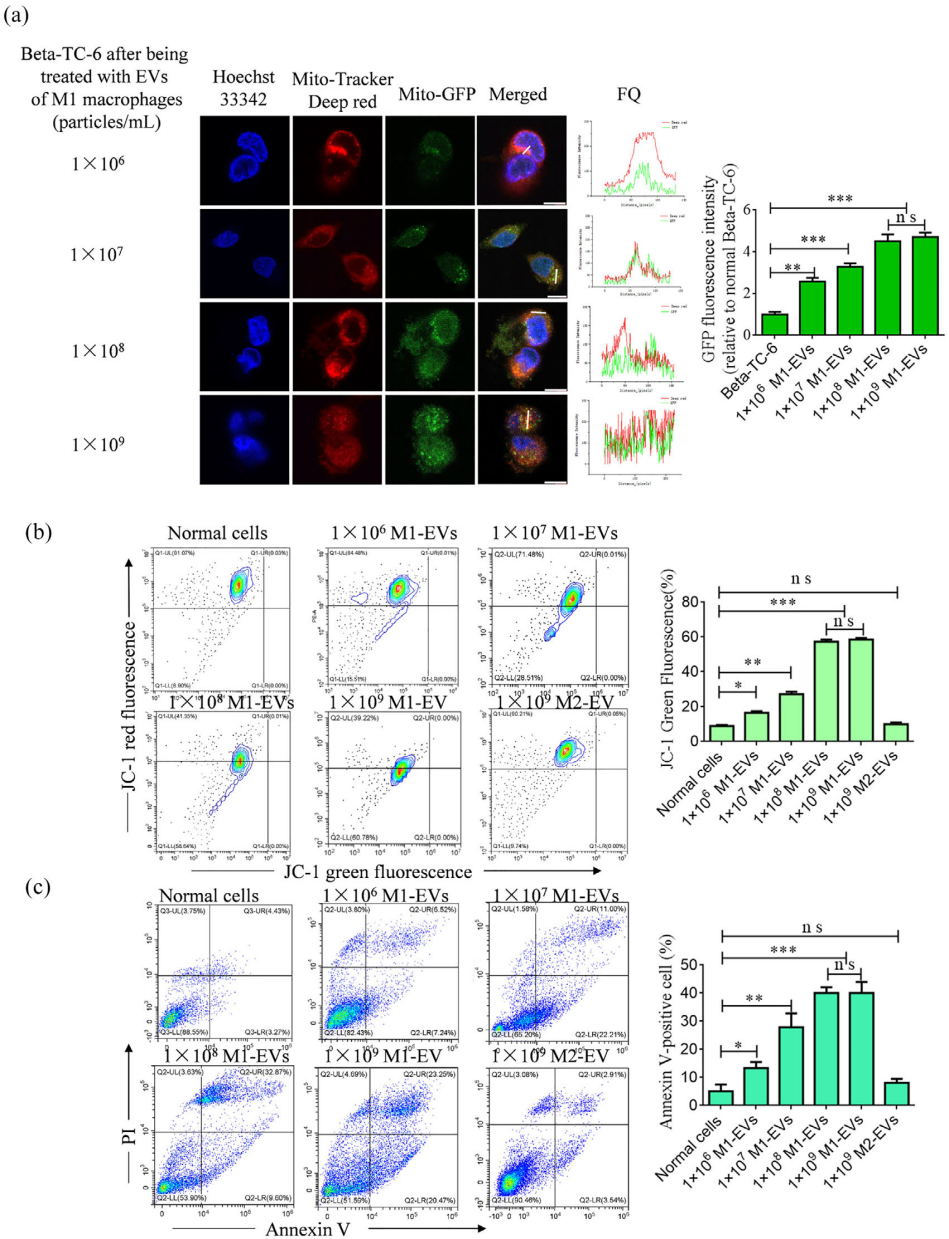
Mitochondria transfer from macrophages to Beta TC‐6 cells through the transportation of extracellular vesicles (EVs). (a) Representative images of mitochondrial transfer from M1 macrophages to Beta TC‐6 cells via transport of EVs. Green fluorescence indicates mitochondria of macrophages, red fluorescence indicates mitochondria of Beta TC‐6 cells, and FQ represents fluorescence quantitation. (b) Analysis of mitochondrial membrane potential in Beta TC‐6 cells after treatment with various concentrations of EVs derived from M1 macrophages or M2 macrophages. (c) Analysis of Annexin X‐positive cells in Beta TC‐6 cells after treatment with various concentrations of EVs derived from M1 macrophages or M2 macrophages. *n* = 3, *P < 0.05, **P < 0.01, ***P < 0.001. ns, not significant.

To investigate the transfer of mitochondria between pancreatic beta cells and macrophages through EV transport, we incubated EVs derived from M1 macrophages labelled with GFP and Beta TC‐6 cells labelled with MitoTracker Deep Red at various concentrations. Following this, we analyzed the co‐localization of fluorescence and found that the results supported the notion of directed mitochondrial transfer. Specifically, we observed green fluorescence in TC‐6 cells, with the intensity of green fluorescence progressively increasing as the concentration of EVs increased, reaching a peak at 1×10^8^ and 1×10^9^ particles/mL. Red fluorescence was also co‐localized with green fluorescence, and the quantification of fluorescence between the red and green signals was observed in the same pixels (Figure 4a). The mitochondrial membrane potential and apoptosis of TC‐6 cells were assessed, and the findings aligned with the concept of directed mitochondrial transfer. A gradual increase in the proportion of apoptotic cells was observed as the concentration of EVs increased in M1 macrophages. This increase reached a maximum at concentrations of 1×10^8^ and 1×10^9^ particles/mL. Conversely, no notable alterations were observed in the mitochondria of M2 macrophages, as evidenced by the analysis of JC‐1 and Annexin V (Figure 4b,c). Therefore, 25 μg/mL M1 mitochondria (M1‐Mito) and 1×10^8^ particles/mL M1‐EVs were used for subsequent experiments.

### 3.3 Inflammatory mitochondria induced apoptosis of beta cells via the ferroptosis pathway

**FIGURE 5:**
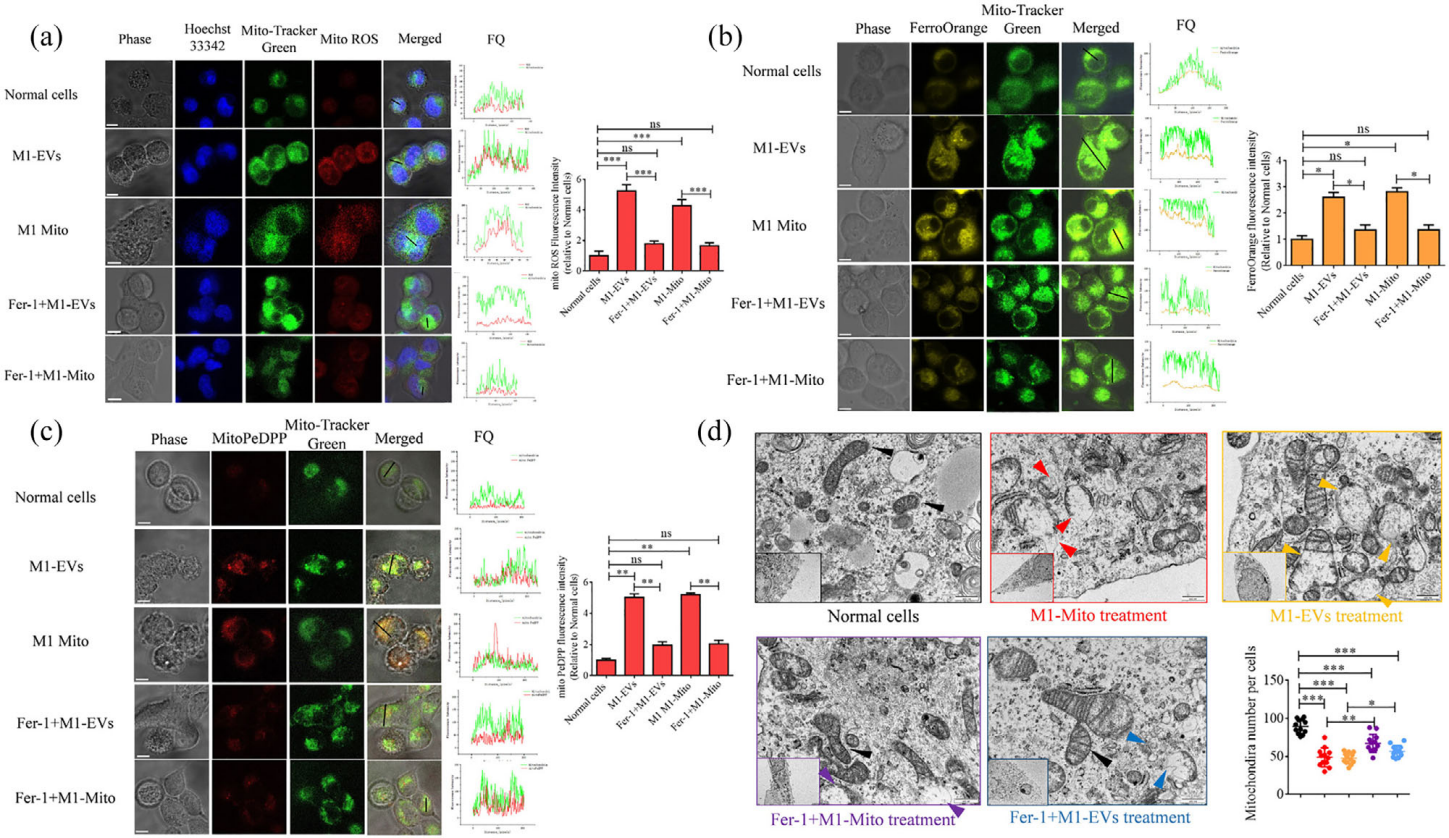
Assessment of mitochondrial reactive oxygen species (mito ROS), ferrous ion (Fe2+) concentration, lipid peroxidation content, and mitochondrial morphology in Beta TC‐6 cells following various treatment protocols. (a) Representative images of mito ROS detection in Beta TC‐6 cells after treatment with M1 mitochondria, M1‐EVs, or a combination of both along with Fer‐1 (*n* = 3). (b) Representative images of assessment of Fe2+ level in Beta TC‐6 cells after different treatment using FerroOrange fluorescence probe (*n* = 3). (c) Representative images of detection of lipid peroxidation content in Beta TC‐6 cells after different treatments using mitoPeDPP fluorescence probe (*n* = 3). (d) Representative images of mitochondrial morphology and quantitative evaluation of mitochondria in Beta TC‐6 cells following various treatments, utilizing a transmission electron microscope (*n* = 10). Red triangle, abnormal mitochondria treated by mitochondria of M1 macrophages; Yellow triangle, abnormal mitochondria treated by EVs derived from M1 macrophages; Purple triangle, abnormal mitochondria treated by mitochondria of M1 macrophages combined with Fer‐1; Blue triangle, abnormal mitochondria treated by EVs derived from M1 macrophages combine with Fer‐1. *P < 0.05, **P < 0.01, ***P < 0.001.

Ferroptosis is an iron‐dependent form of regulated necrosis, and mitochondria play an important role in this process (Gao et al., 2019; Tadokoro et al., 2020). In our study, we initially evaluated ROS, free Fe^2+^ levels, and mitochondria lipid peroxidation in Beta TC‐6 cells following EVs or mitochondria treatment. This assessment was conducted by staining cells with MitoSox Red, FerroOrange, and MitoPeDPP. Our findings revealed significantly higher fluorescence intensities of MitoSox Red, FerroOrange, and MitoPeDPP in EVs or mitochondria‐treated Beta cells compared to normal cells. Furthermore, immunofluorescence localization analysis indicated that the majority of MitoSox Red, FerroOrange, and MitoPeDPPs were localized to the mitochondria. Ferrostatin‐1 (Fer‐1, a ferroptosis inhibitor) was added to M1‐Mito‐ or M1‐EVs‐treated Beta TC‐6 cells to verify the role of ferroptosis, and the fluorescence intensities of MitoSox Red, FerroOrange, and MitoPeDPP dramatically decreased after Fer‐1 addition (Figure 5a‐c). Mitochondrial morphological alterations serve as reliable biomarkers of ferroptosis, a cellular process characterized by augmented mitochondrial permeability and subsequent rupture. In our investigation, we employed transmission electron microscopy to examine the mitochondrial morphology of Beta TC‐6 cells. Normal cells have distinct mitochondrial ridges and intact mitochondrial membranes. Conversely, cells treated with M1‐EVs or M1‐Mito displayed mitochondrial swelling, dissolution of the mitochondrial ridges, and rupture. Additionally, a considerable reduction in the number of mitochondria per cell was observed (Figure 5d).

**FIGURE 6:**
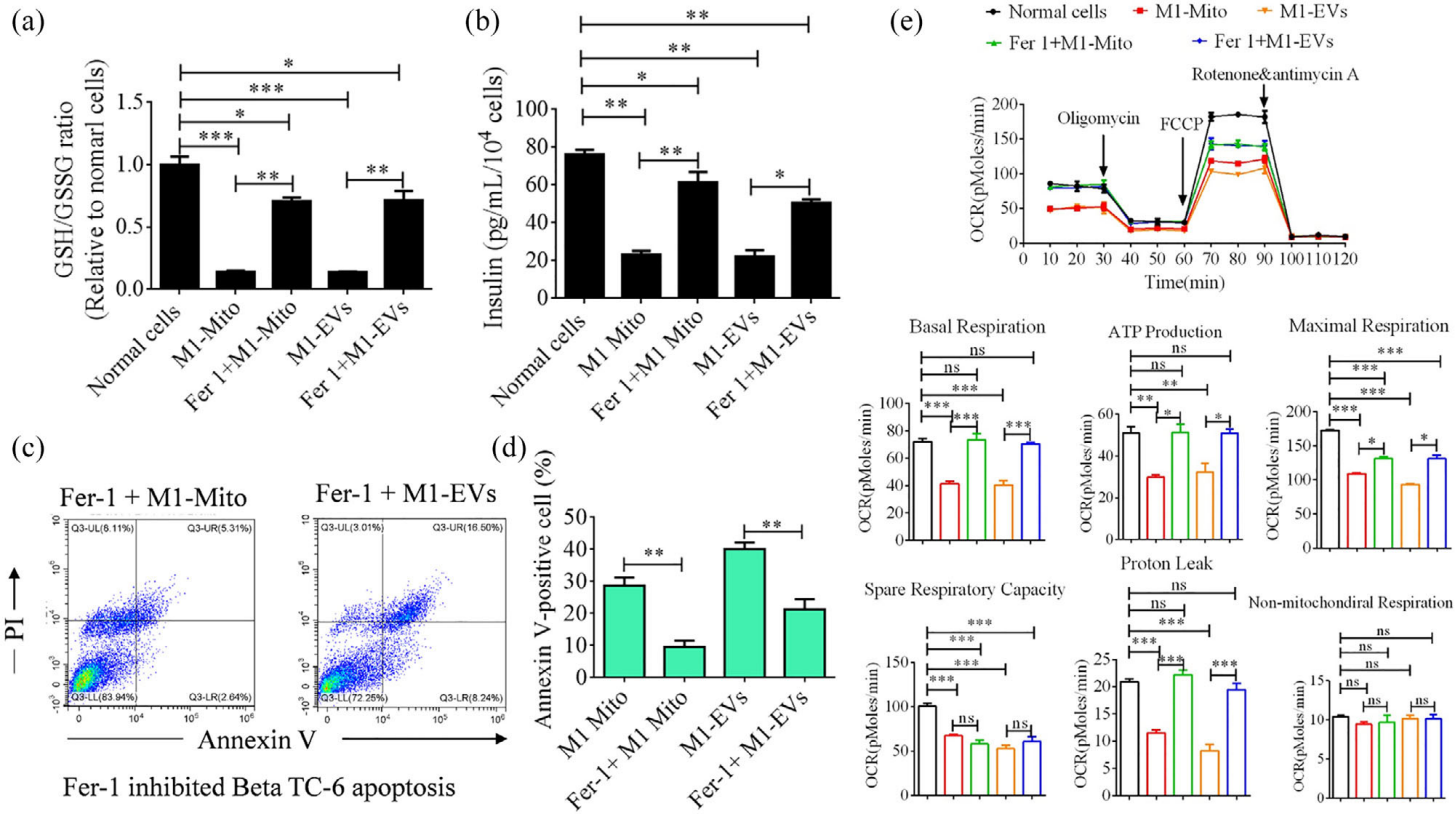
Fer‐1 effectively restored the declining GSH/GSGG ratio, cellular function, and mitochondrial oxygen consumption rate (OCR) in Beta TC‐6 cells following various treatments. (a) Assessment of GSH/GSGG ratio in Beta TC‐6 cells following various treatments. (b) Analysis of insulin secretion from Beta‐TC‐6 cells following various treatments. (c and d) Analysis of Annexin X‐positive cells in Beta TC‐6 cells following various treatments. (e) OCR analysis in Beta TC‐6 cells following various treatments. *n* = 3, *P < 0.05, **P < 0.01, ***P < 0.001. ns, not significant.

GSH/GSSG refers to the ratio of reduced glutathione (GSH) to oxidized glutathione (GSSG) in a biological system and is an important indicator of the cellular redox balance. The GSH/GSSG ratio exhibited a significant decrease following treatment with M1‐EVs or M1‐Mito, compared to that in normal cells. However, this disruption in the current situation was mitigated by the addition of Fer‐1, as depicted in Figure 6a. Subsequently, we investigated the impact of Fer‐1 on insulin secretion and apoptosis in Beta TC‐6 cells. These results corroborated our previous findings, demonstrating that Fer‐1 not only prevented apoptosis but also enhanced insulin secretion in beta cells treated with M1‐EVs or M1‐Mito, as illustrated in Figure 6b‐d. The mitochondrial Oxygen Consumption Rate (OCR) reflects the rate at which mitochondria utilize oxygen within a designated timeframe. In our investigation, we additionally assessed mitochondrial OCR in Beta cells and observed a reduction in mitochondrial ATP production, basal respiration, maximal respiration, spare respiratory capacity, and proton leak subsequent to M1‐EVs or M1‐Mito treatment. However, the inclusion of Fer‐1 mitigated these effects except for maximal respiration and proton leakage (Figure 6e).

### 3.4 Cytosolic mtDNA induces activation of the STING pathway in beta cells

**FIGURE 7:**
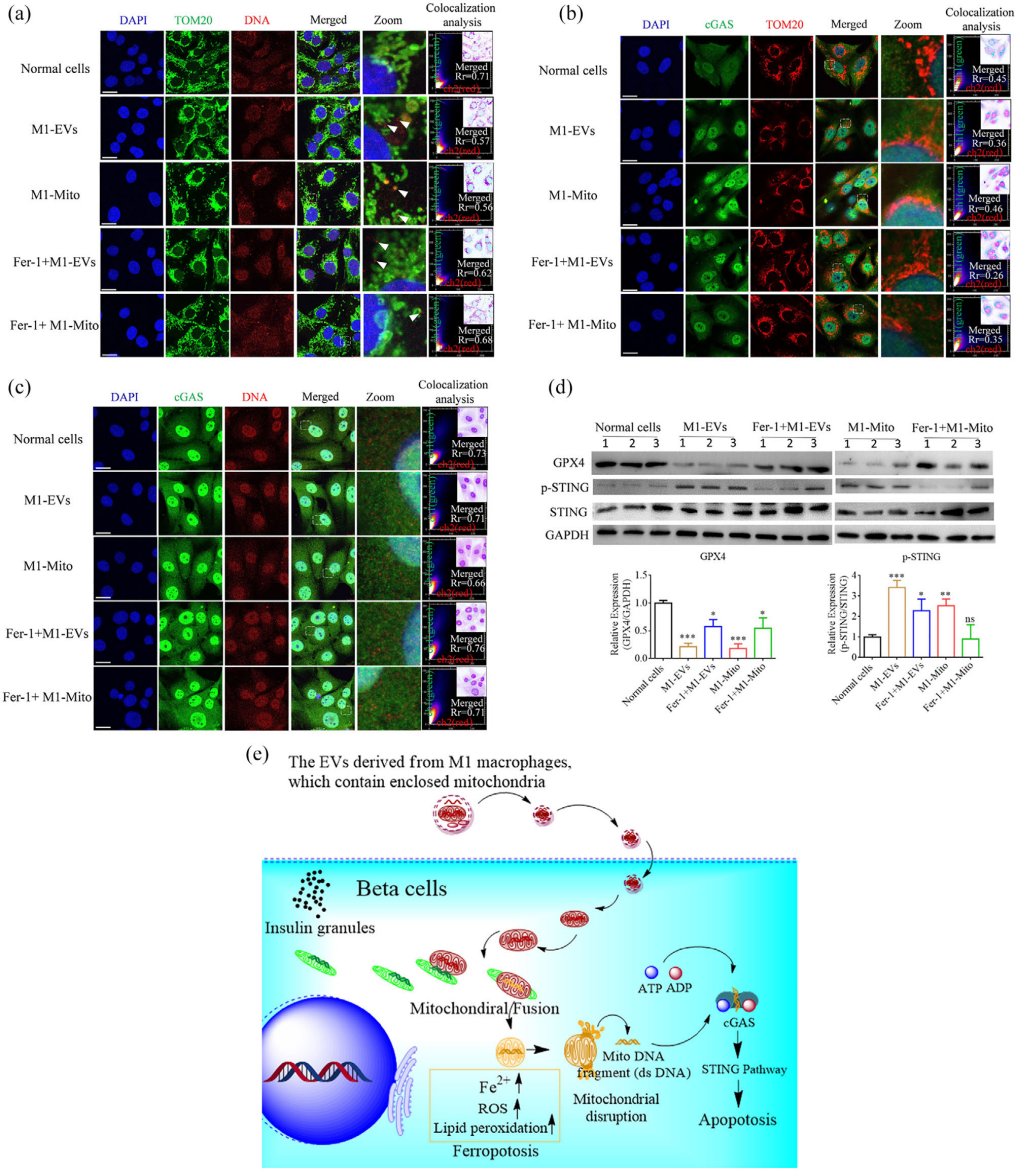
Free mtDNA activated STING pathway in Beta TC‐6 cells following various treatments. (a) Representative images of immunofluorescence staining for mitochondrial marker, TOM20, and dsDNA in Beta TC‐6 cells. (b) Representative images of immunofluorescence staining for mitochondrial marker, TOM20, and cGAS in Beta TC‐6 cells. (c) Representative images of immunofluorescence staining for cGAS and dsDNA in Beta TC‐6 cells. The nucleus was stained with DAPI. Co‐localization analysis of immunofluorescence images using the Image J/colocalization plugin, which calculates Pearson's correlation coefficient. Scale bar = 20 μm. (d) Western blotting was used to analyze the phosphorylation of STING and expression of GPX4. Scale bar = 20 μm. *n* = 3, *P < 0.05, **P < 0.01, ***P < 0.001. (e) Schematic illustration of the mechanism by which M1‐EVs induce apoptosis in beta cells.

The STING pathway is an essential constituent of the innate immune system and plays a pivotal role in the identification of cytosolic DNA and the initiation of an immune response against pathogens. Furthermore, it is associated with cell death pathways, such as apoptosis, pyroptosis, and necroptosis (Motwani et al., 2019; Zhang et al., 2021). Ferroptosis leads to increased mitochondrial permeability and rupture, and mtDNA then accesses the cytosol to form cytosolic DNA (Tadokoro et al., 2020; Zecchini et al., 2023). In the present study, we determined whether the STING pathway was activated by the release of mtDNA caused by ferroptosis. Initially, we demonstrated the release of mtDNA from the mitochondria in TC‐6 cells following treatment with M1‐EVs or M1‐Mito. Immunofluorescence analysis revealed that mtDNA detached from the mitochondrial marker (TOM20) in Beta TC‐6 cells after M1‐EVs or M1‐Mito treatment. Subsequently, co‐localization analysis of the immunofluorescence images was performed using the ImageJ co‐localization plugin tools. Pearson's correlation coefficient (Rr) was calculated to assess the degree of correlation. The results indicated a decline in the correlation coefficient in Beta TC‐6 cells after M1‐EVs or M1‐Mito treatment but an increase upon the addition of Fer‐1 (Figure 7a), implying that Fer‐1 prevented mitochondrial rupture. Cyclic GMP‐AMP synthase (cGAS), initially identified as a double‐stranded DNA sensor, plays a vital role in the innate immune response against pathogens (Sun et al., 2013; Tadokoro et al., 2023). To investigate the interaction between cGAS and mitochondria, co‐localization analysis was conducted using immunofluorescence images, focusing on the co‐localization of TOM20 with cGAS in TC‐6 cells after different treatments. The obtained Pearson's correlation coefficient (Rr) was below 5.0, indicating the absence of co‐localization between the mitochondria and cGAS (Figure 7b). Subsequent evaluation involved examining the interaction between cGAS and cytosolic mtDNA, which revealed its presence in TC‐6 cells following various treatments (Figure 7c). To examine the necessity of the interaction between cGAS and cytosolic mtDNA for the activation of the STING pathway in TC‐6 cells under different treatments, we used western blotting to assess the expression of GPX4 and the phosphorylation levels of STING and IRF3. The findings revealed the downregulation of GPX4, along with an increase in the phosphorylation of both STING and IRF3 in Beta TC‐6 cells treated with M1‐EVs or M1‐Mito. The addition of Fer‐1 yielded contrasting results (Figure 7d and Figure S7). In summary, M1 macrophages employ EVs to transport mitochondria to pancreatic beta cells. The fusion of these mitochondria with those already present in the beta cells induces the accumulation of Fe^2+^ within the cell, augmenting mitochondrial reactive oxygen species (ROS) and lipid peroxidation. Consequently, mitochondrial rupture occurs, followed by the release of mtDNA into the cytoplasm. Subsequently, the STING pathway was activated, ultimately culminating in cell death (Figure 7e). In summary, the release of EVs by M1 macrophages facilitated the death of pancreatic beta cells. Consequently, addressing the death of pancreatic beta cells caused by M1 macrophages necessitates a concentrated approach to impede M1 macrophage polarization. By effectively inhibiting the polarization of M1 macrophages, it is possible to mitigate glucose metabolism abnormalities induced by AP.

### 3.5 Surface modification of ADSC‐derived EVs

**FIGURE 8:**
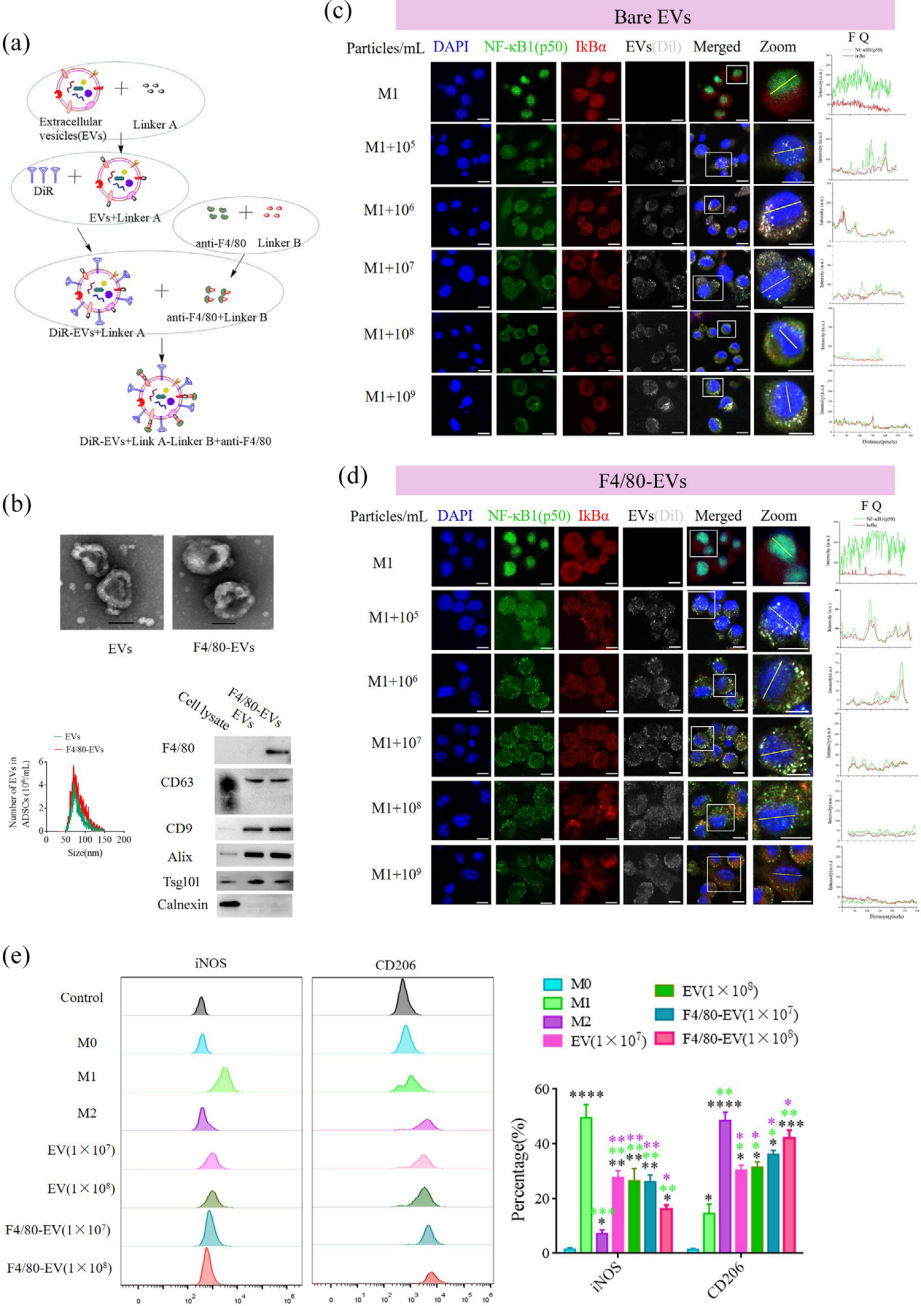
Surface modification of ADSC‐derived EVs and converting macrophage polarization in vitro. (a) Schematic diagram of the anti‐F4/80 antibody decorated on the surface of ADSC‐derived EVs. (b) Biological characteristics of F4/80‐EVs, including morphology, particulate size distribution, and EV marker proteins. The data indicated no significant change in morphology, particulate size distribution, or EV marker proteins but excluded F4/80 in normal EVs. To assess the integration of the rat anti‐F4/80 antibody with EVs, the lysates were immunoblotted with HRP‐conjugated donkey anti‐rat IgG. The results were positive in the F4/80‐EVs group and negative in the normal EVs group. (c) Various concentrations of ADSC‐secreted EVs (bare EVs) were incubated with M1 phenotype macrophages. (d) Various concentrations of anti‐F4/80‐decorated, ADSC‐secreted EVs (F4/80‐EVs) were incubated with M1 phenotype macrophages. Fluorescence images were used to evaluate the localization of FITC‐labelled NF‐κB1 (p50) and Cy5‐labelled IκBα. FITC fluorescence signals were initially observed in the nucleus, and after incubation with EVs, the FITC signals were co‐localized with Cy5 in the cytoplasm. Fluorescence quantitation of arbitrary single cells revealed fewer FITC signals in the F4/80‐EV group than in the EV group at the same concentration. FQ, fluorescence quantitation; a.u., arbitrary unit. (e) Flow cytometry of M1 (iNOS) and M2 (CD206) macrophage markers in macrophages after treatment with various concentrations (left) and quantification of iNOS and CD206 expression levels in EV and F4/80‐EV‐treated macrophages (*n* = 3). Black superscript, M0 versus each group; Laurel green superscript, M1 versus each group; Purple superscript, M2‐EVs versus each group. *P < 0.05, **P < 0.01, ***P < 0.001.

The reversal of the macrophage phenotype from M1 to M2 is achieved through the secretion of EVs by MSCs. Consequently, EVs derived from ADSCs have been utilized to counteract the proinflammatory phenotype of macrophages, thereby preventing the death of pancreatic beta cells both in vitro and in vivo. First, the biological characteristics of human ADSCs, including specific marker expression and multi‐differentiation potential, were identified. Our results demonstrate that human ADSCs were positive for CD29, CD90, and CD105 and that the ADSCs successfully differentiated into chondrocytes, adipocytes, and osteoblasts (Figure S8). Previous reports (W. Liu et al., 2021; You et al., 2021) have shown that EVs derived from MSCs carry certain miRNAs and proteases that reverse the dominant phenotype from M1 to M2 in macrophages, and the reverse effect of EVs secreted from MSCs pretreated with melatonin is dramatically elevated. In this study, the levels of specialized miRNAs (let‐7b‐5p and miR‐24‐3p) and a protease (USP29) were markedly elevated in the EVs derived from melatonin‐pretreated ADSCs (Figure S9A and B), which facilitated M2 polarization. These EVs were similar in terms of particle size distribution, spherical shape, and EV marker proteins before and after melatonin treatment (Figures S9B–D, and S10) and significantly elevated the percentage of CD206‐positive cells in M1 macrophages (Figure S9E–H). Therefore, EVs derived from melatonin‐pretreated ADSCs were used in subsequent experiments. To improve the delivery efficiency of EVs in vivo, activated macrophage‐targeting ADSC‐derived EVs were prepared, and a rat monoclonal against F4/80 antibody was decorated on the surface of the ADSC‐derived EVs using click chemistry (called F4/80‐EVs, Figure 8a). The biological characteristics of F4/80‐EVs and normal EVs were analyzed, and the data indicated no significant changes in morphology, particulate size distribution, or EV marker proteins, excluding F4/80 in normal EVs (Figure 8b and Figure S11). NF‐κB is a dimer of members of the Rel family consisting of a group of five proteins. A heterodimer of NF‐κB (p65) and NF‐κB1 (p50) subunits, which was the first described NF‐κB molecule, is inhibited by IκBα protein in unstimulated cells. p65 and p50 were detected via immunofluorescence staining in macrophages after M1 polarization (Figure S12). p50 had almost entered the nucleus, whereas some p65 remained in the cytoplasm. In order to examine the impact of F4/80‐EVs on macrophage polarization, we performed in vitro immunofluorescence staining and flow cytometry analyses on macrophages. These analyses aimed to assess the expression of NF‐κB1 (p50) in the nucleus, as well as the percentage changes in iNOS and CD206 within macrophages following various treatments conducted under identical experimental conditions. Immunofluorescence showed that NF‐κB1 (p50) was effectively restricted to the cytoplasm after EV (Figure 8c) and F4/80‐EV (Figure 8d) treatments in a concentration‐dependent manner under. FITC‐labelled NF‐κB1 (p50) and Cy5‐labelled IκBα were used to evaluate localization. FITC fluorescence signals were initially observed in the nucleus; however, after incubation with EVs, FITC signals co‐localized with Cy5 in the cytoplasm. The fluorescence quantification of arbitrary single cells revealed fewer FITC signals in the F4/80‐EV group than in the EV group at the same concentrations (Figure S13A). The expression levels of iNOS, a marker of M1 macrophages, and CD206, a marker of M2 macrophages, were analyzed using flow cytometry. The results revealed that the percentage of CD206‐positive cells in the F4/80‐EV group was significantly higher than in the EV group at the same concentration (Figure 8e). The inflammatory factors, interleukin (IL)−1β, tumour necrosis factor (TNF)‐α, and IL‐6, were identified in both the transcription and secretion processes within M1 macrophages and cell suspension. This was achieved using real‐time PCR and ELISA. The accuracy of these findings was further validated using data obtained from the immunofluorescence and flow cytometry analyses, which are presented in Figure S13B and C. These data imply that surface modification of EVs by the anit‐F4/80 antibody conferred better properties for converting the macrophage phenotype from M1 to M2 in vitro.

### 3.6 Targeted delivery F4/80‐EVs to macrophages in vivo and its therapeutic efficacy in AP mice

**FIGURE 9:**
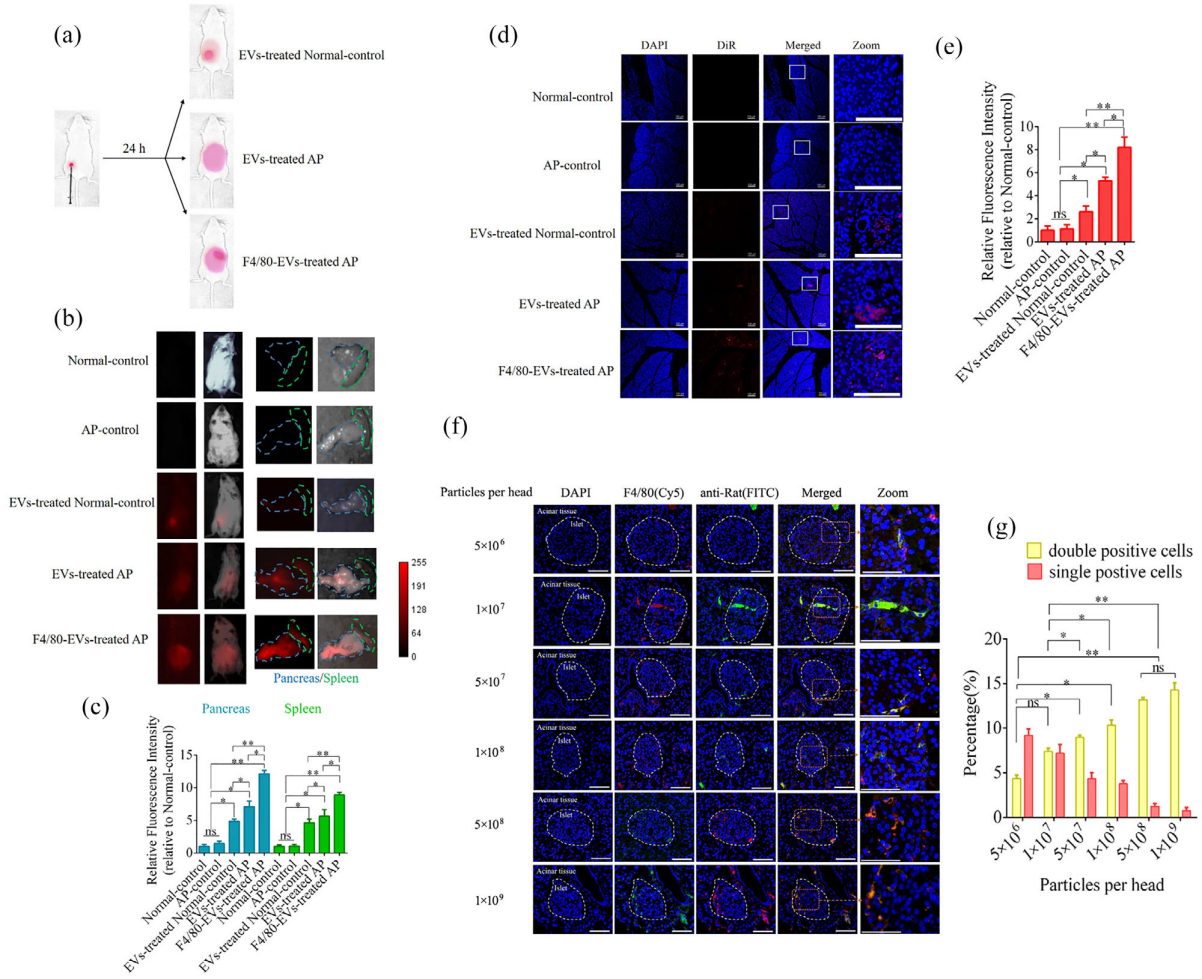
Targeted delivery of F4/80‐EVs to macrophages in vivo. (a) Schematic diagram of the effects of targeted delivery of F4/80‐EVs in AP mice. (b) Representative whole body and organ fluorescence imaging of EVs and F4/80‐EVs (1×109 particles per mouse) in AP and normal mice. (c) Relative fluorescence intensity of EVs and F4/80‐EVs in the pancreas and spleen of normal and AP mice (*n* = 3). (d) Representative fluorescence imaging of pancreatic tissue sections. Scale bar = 100 μm. (e) Relative fluorescence intensity of EVs and F4/80‐EVs in the pancreas of normal and AP mice. (f) Location of F4/80‐EVs in pancreas sections from AP mice using the corresponding secondary antibody (labelled FITC) of F4/80. A rabbit polyclonal antibody against F4/80 labelled with Cy5 was used to verify macrophages. Double‐positive cells indicated macrophages that carried F4/80‐EVs. (g) Quantification of double‐positive cells in pancreas section of AP mice after treatment with various concentrations of F4/80‐EVs. *n* = 3. ns, not significant, *P < 0.05, **P < 0.01, ***P < 0.001.

To further verify the F4/80‐mediated endocytosis of F4/80‐EVs by macrophages in the pancreas, normal and AP mice were used to assess targeted delivery by intraperitoneal injection of 1×10^9^ particles of EVs or F4/80‐EVs per mouse. Whole‐body fluorescence imaging was used to analyze the location of EVs. As shown in Figure 9a,b, the majority of fluorescence signals remained at the injection site of normal mice 24 h after EV treatment. Conversely, the fluorescence signals spread to the abdomen in EV‐treated AP mice, and the pancreas showed stronger fluorescence signals than those in AP mice treated with F4/80‐EVs. Next, the organs of these mice were harvested to assess EV distribution in vivo. Compared to normal mice, EVs and F4/80‐EVs accumulated mostly in the pancreas and spleen, with some distribution in the gut, kidney, and liver of AP mice (Figure 9b and Figure S14). The fluorescence intensity of F4/80‐EVs in the pancreas and spleen was significantly higher than that of EVs (Figure 9c). Next, we examined the distribution of fluorescence signals using laser scanning confocal microscopy of pancreatic sections from these mice. The results closely aligned with those of whole‐body and organ imaging. Fluorescence was observed in the pancreas of AP mice after EV and F4/80‐EV treatment; however, the fluorescence in the F4/80‐EV group was significantly higher than that in the EV treatment group (Figure 9d,e). To verify the targeted delivery of F4/80‐EVs to macrophages in vivo, a rabbit polyclonal antibody against F4/80 labelled with Cy5 was used to locate macrophages in pancreatic sections, and an anti‐rat antibody labelled with FITC was used to locate the rat polyclonal antibody against F4/80, which decorated the EV surface. Double‐positive cells indicated macrophages that contained F4/80‐EVs, which increased with F4/80‐EV concentration. Double‐positive cells in pancreatic sections increased gradually, peaking at 5×10^8^ and 1×10^9^ particles per mouse (Figure 9f,g).

**FIGURE 10:**
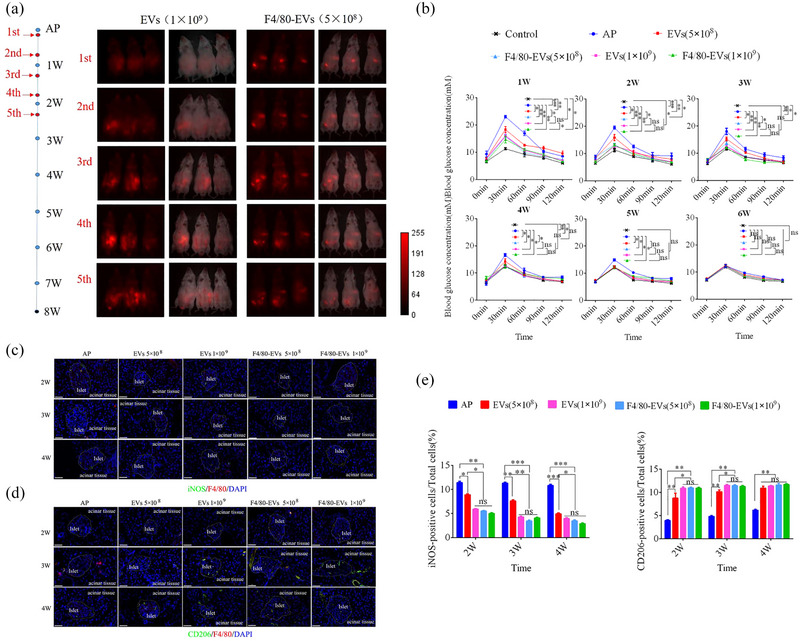
Therapeutic efficacy of EVs and F4/80‐EVs in AP mice. (a) Treatment regimen used for EVs and F4/80‐EVs (left). Red points represent intraperitoneal injection of samples. Representative whole‐body fluorescence imaging of EVs and F4/80‐EVs in AP mice (right). Fluorescence images were obtained after 24 h of treatment with EVs and F4/80‐EVs. (b) Improvement of abnormal glucose metabolism in AP mice by various concentrations of EVs and F4/80‐EVs. (c and d) Representative immunofluorescence images of iNOS and CD206‐positive cells in the pancreas of variously treated AP mice. (e) Quantification of iNOS and CD206‐positive cells in pancreatic tissue at various time points after various treatments of AP mice. *n* = 3, *P < 0.05, **P < 0.01, ***P < 0.001, ns, not significant.

To investigate the effects of F4/80‐EVs and EVs on macrophage polarization and their therapeutic efficacy in vivo, based on a previous report (You et al., 2021), we systemically administered EVs via five intraperitoneal injections at an interval of 3 days into AP mice. Whole‐body fluorescence imaging was performed 24 h after the treatment using a live imaging system (Figure 10a). The fluorescence signals became more concentrated in the pancreas and injection sites as the F4/80‐EV dose increased. The glucose tolerance test showed significant improvement in all EV treatment groups. The F4/80‐EV group at 1×10^9^ particles per mouse was the first to recover to the normal level after 3 weeks, whereas recovery occurred after 4 weeks in the F4/80‐EV group at 5×10^8^ particles per mouse and in the EV group at 1×10^9^ particles per mouse (Figure 10b). To further validate the inhibitory impact of each group treated with EV on the inflammatory response, the levels of inflammatory factors, namely IL‐1β, TNF‐α, and IL‐6, were assessed in plasma using ELISA. The results indicate a significant reduction in the levels of inflammatory factors following EV treatment. Notably, the F4/80‐EV group at a dosage of 1×10^9^ particles per mouse, the F4/80‐EV group at a dosage of 5×10^8^ particles per mouse, and the F4/80‐EV group at a dosage of 1×10^9^ particles per mouse exhibited superior efficacy than did the EV group at a dosage of 5×10^8^ particles per mouse. Furthermore, the HOMA‐β analysis corroborated the aforementioned findings (Figure S15).

**FIGURE 11:**
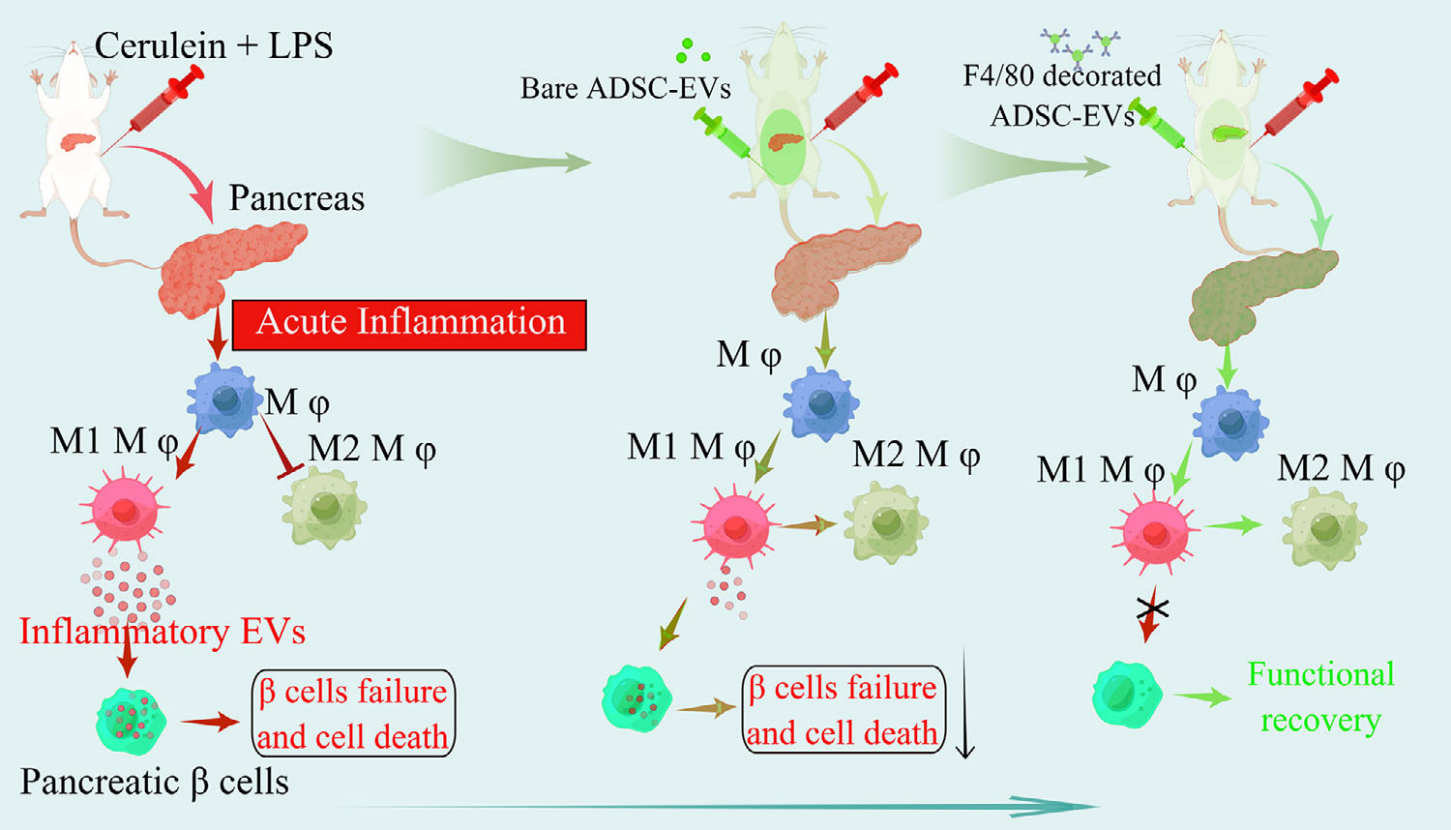
Schematic diagram illustrating the therapeutic impact of ADSC‐EVs and F4/80 antibody‐modified ADSC‐EVs on the aberrant glucose metabolism of an AP model mouse. ADSC, adipose‐derived stem cell; LPS, lipopolysaccharide; EVs, extracellular vesicles.

Next, we analyzed macrophage polarization in AP mice using anti‐iNOS and ‐CD206 antibodies after 2, 3, and 4 weeks of treatment with EVs or F4/80‐EVs. The results demonstrated that the percentage of CD206‐positive cells was highest in the F4/80‐EV group at 1×10^9^ particles per mouse compared to the other groups. Additionally, the percentage of CD206‐positive cells in the F4/80‐EV group at 5×10^8^ particles per mouse was almost equal to that in the EV group at 1×10^9^ particles per mouse (Figure 10c‐e). Consistent with the therapeutic efficacy, the F4/80‐EV group at 5×10^8^ particles per mouse converted M1 to M2 polarization in vivo, similar to the EV group at 1 × 10^9^ particles per mouse (Figure 11).

## 4 DISCUSSION

Ferroptosis is linked to multiple cellular metabolic pathways such as redox homeostasis, iron metabolism, mitochondrial activity, and various disease‐related signalling pathways (Dixon et al., 2012; Gao et al., 2019; Q. Li et al., 2017; Stockwell et al., 2017). Recent research has demonstrated notable impacts of ferroptosis on the advancement and course of type 1 diabetes mellitus (Krummel et al., 2021). In pancreatic beta cells, the heightened expression of GPX4 hinders cell death via the apoptotic pathway instead of ferroptosis when influenced by proinflammatory factors. However, when the ROS levels increase, the expression of GPX4 is diminished, resulting in cell death. The mitochondria play a pivotal role in ferroptosis by actively contributing to the release of intracellular iron ions (Gao et al., 2019; Lin et al., 2022). In the context of ferroptosis, the iron within the mitochondria is released into the cytoplasm via specialized transport proteins, such as transferrin. This release amplifies lipid peroxidation and oxidative stress, ultimately culminating in cell death (Gao et al., 2019). Previous studies have indicated that macrophages serve as the source of transferred mitochondria, exerting regulatory control over the inflammatory response in myocardial and neuronal tissues (J. Chen, Fu et al., 2022a; van der Vlist et al., 2022). Macrophages facilitate the transfer of mitochondria to cardiomyocytes, thereby inducing cardiomyocyte injury through the activation of ferroptosis. The mechanisms involved in this process include both direct transfer and EV‐mediated transfer. In a study conducted by D'Souza et al., it was observed that EVs derived from human brain endothelial cells were capable of transferring mitochondria, leading to enhanced survival of endothelial cells during ischemic conditions (D'Souza et al., 2021). This provides evidence that EVs derived from cancer‐associated fibroblasts, which contain intact mitochondrial genome DNA, function as an oncogenic signal, facilitating the emergence of therapy‐induced cancer stem‐like cells from a dormant state and consequently contribute to the development of resistance to endocrine therapy in breast cancer (Sansone et al., 2017). To date, there have been no reports on the induction of ferroptosis in pancreatic beta cells by M1 macrophages through the transportation of mitochondria via EVs. In this study, we successfully illustrated that EVs derived from M1 macrophages possess the ability to transport inflammatory mitochondria into beta cells. Upon fusion with resident mitochondria, this process triggers the ferroptosis pathway, leading to mitochondrial disruption and the subsequent release of mtDNA into the cytoplasm. Subsequently, the liberated mtDNA activates the STING pathway, ultimately culminating in apoptosis.

Humans with AP often develop prediabetes and diabetes mellitus after recovery and have a greater than two‐fold increased risk of diabetes mellitus over 5 years (Das et al., 2014; Petrov & Yadav, 2019). Therefore, inhibiting the polarization of macrophages towards a proinflammatory phenotype during pancreatitis and preventing the release of their proinflammatory EVs are new targets for the prevention or treatment of PPDM. EVs secreted from ADSCs have emerged as potential therapeutics because of their anti‐inflammatory effects on bioactive molecules such as miRNAs and proteases, which modulate the biological activities of recipient cells. ADSC‐secreted EVs containing specialized miRNAs facilitate the conversion of M1 phenotype macrophages to the M2 phenotype in vivo after surface modification for targeted delivery to macrophages for therapy of rheumatoid arthritis (You et al., 2021). Additionally, Liu et al. demonstrated that these EVs carried ubiquitin‐specific proteases secreted from MSCs to increase the expression of nuclear factor‐like 2 (NRF2) in microglia/macrophages after EV endocytosis and then converted microglia/macrophage polarization from the M1 phenotype to the M2 phenotype for therapy of spinal cord injury (W. Liu et al., 2021). In this study, to further determine the role of proinflammatory macrophages in the death of beta cells in vivo, human ADSC‐secreted EVs were used to prevent the activation of the NF‐κB pathway and convert the macrophage phenotype from M1 to M2 in vitro. Subsequently, an anti‐F4/80 antibody was decorated on the surface of the EVs that were targeted for delivery to macrophages for therapy in an AP mouse model. We obtained the ideal result that glycometabolism recovered after F4/80‐EV treatment, which implied that proinflammatory macrophages play a major role in beta cell failure and apoptosis during PPDM progression.

In conclusion, we examined the pathogenesis of PPDM in a mouse model. Our analyses revealed that EVs derived from M1 phenotype macrophages possess the ability to transport inflammatory mitochondria into beta cells and induce beta cell death through ferroptosis and STING pathways. ADSC‐secreted EVs were decorated with an anti‐F4/80 antibody for targeted delivery to macrophages in vivo for abnormal glucose therapy in AP. Our results further demonstrated that M1 phenotype macrophages play a major role in beta cell failure and apoptosis, and the modified ADSC‐EVs display considerable potential for development as a vehicle for the targeted delivery of PPDM.

## AUTHOR CONTRIBUTIONS

**Yuhua Gao**: Conceptualization; investigation; methodology; visualization; writing—original draft. **Ningning Mi**: Investigation; methodology; visualization; writing—original draft. **Wenxiang Wu**: Investigation; methodology. **Yuxuan Zhao**: Investigation; methodology. **Fangzhou Fan**: Investigation; methodology. **Wangwei Liao**: Investigation; methodology. **Yongliang Ming**: Investigation; methodology. **Weijun Guan**: Conceptualization; funding acquisition; resources. **Chunyu Bai**: Conceptualization; funding acquisition (lead); methodology; supervision; writing—original draft; writing—review and editing.

## CONFLICT OF INTEREST STATEMENT

The authors declare no conflicts of interest.

## Supporting information

Supplementary Information

Supplementary Information

Supplementary Information

## Data Availability

The raw data used to support the conclusions of this article will be made available by the corresponding authors, without undue reservation, to any qualified researcher. The mass spectrometry proteomics data have been deposited to the ProteomeXchange Consortium (http://proteomecentral.proteomexchange.org) via the iProX partner repository (T. Chen, Ma et al., 2022; Ma et al., 2019) with the dataset identifier PXD044444.

## References

[jev212410-bib-0001] Alonso‐Herranz, L. , Porcuna, J. , & Ricote, M. (2019). Isolation and purification of tissue resident macrophages for the analysis of nuclear receptor activity. Methods in Molecular Biology, 1951, 59–73. 30825144 10.1007/978-1-4939-9130-3_5

[jev212410-bib-0002] Cai, W. J. , Zhang, J. L. , Yu, Y. Q. , Ni, Y. Q. , Wei, Y. , Cheng, Y. H. , Han, L. , Xiao, L. , Ma, X. , Wei, H. , Ji, Y. , & Zhang, Y. (2023). Mitochondrial transfer regulates cell fate through metabolic remodeling in osteoporosis. Advancement of Science, 10(4), e2204871. 10.1002/advs.202204871 PMC9896036 36507570

[jev212410-bib-0003] Chen, J. , Fu, C. Y. , Shen, G. , Wang, J. , Xu, L. , Li, H. , Cao, X. , Zheng, M.‐Z. , Shen, Y.‐L. , Zhong, J. , Chen, Y.‐Y. , & Wang, L. L. (2022a). Macrophages induce cardiomyocyte ferroptosis via mitochondrial transfer. Free Radical Biology and Medicine, 190, 1–14. 35933052 10.1016/j.freeradbiomed.2022.07.015

[jev212410-bib-0004] Chen, J. , Zhong, J. J. , Wang, L. L. , & Chen, Y. Y. (2021). Mitochondrial transfer in cardiovascular disease: From mechanisms to therapeutic implications. Frontiers in Cardiovascular Medicine, 8, 771298. 34901230 10.3389/fcvm.2021.771298 PMC8661009

[jev212410-bib-0005] Chen, T. , Ma, J. , Liu, Y. , Chen, Z. , Xiao, N. , Lu, Y. , Fu, Y. , Yang, C. , Li, M. , Wu, S. , Wang, X. , Li, D. , He, F. , Hermjakob, H. , & Zhu, Y. (2022b). iProX in 2021: Connecting proteomics data sharing with big data. Nucleic Acids Research., 50, D1522–D1527. 34871441 10.1093/nar/gkab1081 PMC8728291

[jev212410-bib-0006] Das, S. L. , Singh, P. P. , Phillips, A. R. , Murphy, R. , Windsor, J. A. , & Petrov, M. S. (2014). Newly diagnosed diabetes mellitus after acute pancreatitis: A systematic review and meta‐analysis. Gut, 63, 818–831. 23929695 10.1136/gutjnl-2013-305062

[jev212410-bib-0007] Ding, H. , Li, L. D. X. Y. , Harris, P. C. , Yang, J. W. , & Li, X. G. (2021). Extracellular vesicles and exosomes generated from cystic renal epithelial cells promote cyst growth in autosomal dominant polycystic kidney disease. Nature Communications, 12, 4548. 10.1038/s41467-021-24799-x PMC8316472 34315885

[jev212410-bib-0008] Dixon, S. J. , Lemberg, K. M. , Lamprecht, M. R. , Skouta, R. , Zaitsev, E. M. , Gleason, C. E. , Patel, D. N. , Bauer, A. J. , Cantley, A. M. , Yang, W. S. , Morrison, B. , & Stockwell, B. R. (2012). Ferroptosis: An iron‐dependent form of nonapoptotic cell death. Cell, 149, 1060–1072. 22632970 10.1016/j.cell.2012.03.042 PMC3367386

[jev212410-bib-0009] D'Souza, A. , Burch, A. , Dave, K. M. , Sreeram, A. , Reynolds, M. J. , Dobbins, D. X. , Kamte, Y. S. , Zhao, W. , Sabatelle, C. , Joy, G. M. , Soman, V. , Chandran, U. R. , Shiva, S. S. , Quillinan, N. , Herson, P. S. , & Manickam, D. S. (2021). Microvesicles transfer mitochondria and increase mitochondrial function in brain endothelial cells. Journal of Controlled Release, 338, 505–526. 34450196 10.1016/j.jconrel.2021.08.038 PMC8526414

[jev212410-bib-0010] Essandoh, K. , Yang, L. , Wang, X. , Huang, W. , Qin, D. , Hao, J. , Wang, Y. , Zingarelli, B. , Peng, T. , & Fan, G. C. (2015). Blockade of exosome generation with GW4869 dampens the sepsis‐induced inflammation and cardiac dysfunction. Biochimica Et Biophysica Acta, 1852, 2362–2371. 26300484 10.1016/j.bbadis.2015.08.010 PMC4581992

[jev212410-bib-0011] Gao, M. , Yi, J. , Zhu, J. , Minikes, A. M. , Monian, P. , Thompson, C. B. , & Jiang, X. (2019). Role of mitochondria in ferroptosis. Molecular Cell, 73, 354–363.e353. 30581146 10.1016/j.molcel.2018.10.042 PMC6338496

[jev212410-bib-0012] Guay, C. , Kruit, J. K. , Rome, S. , Menoud, V. , Mulder, N. L. , Jurdzinski, A. , Mancarella, F. , Sebastiani, G. , Donda, A. , Gonzalez, B. J. , Jandus, C. , Bouzakri, K. , Pinget, M. , Boitard, C. , Romero, P. , Dotta, F. , & Regazzi, R. (2019). Lymphocyte‐derived exosomal microRNAs promote pancreatic beta cell death and may contribute to Type 1 diabetes development. Cell metabolism, 29, 348–361.e346. 30318337 10.1016/j.cmet.2018.09.011

[jev212410-bib-0013] Krummel, B. , Plotz, T. , Jorns, A. , Lenzen, S. , & Mehmeti, I. (2021). The central role of glutathione peroxidase 4 in the regulation of ferroptosis and its implications for pro‐inflammatory cytokine‐mediated beta‐cell death. Biochimica et Biophysica Acta: Molecular Basis of Disease, 1867, 166114. 33662571 10.1016/j.bbadis.2021.166114

[jev212410-bib-0014] Li, H. Y. Z. , Wang, C. , He, T. , Zhao, T. F. , Chen, Y. Y. , Shen, Y. L. , Zhang, X. M. , & Wang, L. L. (2019). Mitochondrial transfer from bone marrow mesenchymal stem cells to motor neurons in spinal cord injury rats via gap junction. Theranostics, 9, 2017–2035. 31037154 10.7150/thno.29400 PMC6485285

[jev212410-bib-0015] Li, J. , Liu, K. , Liu, Y. , Xu, Y. , Zhang, F. , Yang, H. , Liu, J. , Pan, T. , Chen, J. , Wu, M. , Zhou, X. , & Yuan, Z. (2013). Exosomes mediate the cell‐to‐cell transmission of IFN‐alpha‐induced antiviral activity. Nature Immunology, 14, 793–803. 23832071 10.1038/ni.2647

[jev212410-bib-0016] Li, Q. , Han, X. , Lan, X. , Gao, Y. , Wan, J. , Durham, F. , Cheng, T. , Yang, J. , Wang, Z. , Jiang, C. , Ying, M. , Koehler, R. C. , Stockwell, B. R. , & Wang, J. (2017). Inhibition of neuronal ferroptosis protects hemorrhagic brain. JCI Insight, 2, e90777. 28405617 10.1172/jci.insight.90777 PMC5374066

[jev212410-bib-0017] Lin, Y. , Zhang, H. , Chen, H. , Pauklin, S. , & Zeng, Q. (2022). Editorial: The role of mitochondria and ferroptosis in cell fate. Frontiers in Cell and Developmental Biology, 10, 1025709. 36506098 10.3389/fcell.2022.1025709 PMC9732848

[jev212410-bib-0018] Liu, C. , Hu, F. , Jiao, G. , Guo, Y. , Zhou, P. , Zhang, Y. , Zhang, Z. , Yi, J. , You, Y. , Li, Z. , Wang, H. , & Zhang, X. (2022a). Dental pulp stem cell‐derived exosomes suppress M1 macrophage polarization through the ROS‐MAPK‐NFkappaB P65 signaling pathway after spinal cord injury. Journal of Nanobiotechnology, 20, 65. 35109874 10.1186/s12951-022-01273-4 PMC8811988

[jev212410-bib-0019] Liu, W. , Tang, P. , Wang, J. , Ye, W. , Ge, X. , Rong, Y. , Ji, C. , Wang, Z. , Bai, J. , Fan, J. , Yin, G. , & Cai, W. (2021). Extracellular vesicles derived from melatonin‐preconditioned mesenchymal stem cells containing USP29 repair traumatic spinal cord injury by stabilizing NRF2. Journal of Pineal Research, 71, e12769. 34562326 10.1111/jpi.12769

[jev212410-bib-0020] Liu, Y. H. , Wu, M. Y. , Zhong, C. X. , Xu, B. , & Kang, L. N. (2022b). M2‐like macrophages transplantation protects against the doxorubicin‐induced heart failure via mitochondrial transfer. Biomaterials Research, *26*. 10.1186/s40824-022-00260-y PMC8996664 35410296

[jev212410-bib-0021] Liu, Z. , Gan, L. , Zhang, T. , Ren, Q. , & Sun, C. (2018). Melatonin alleviates adipose inflammation through elevating alpha‐ketoglutarate and diverting adipose‐derived exosomes to macrophages in mice. Journal of Pineal Research, *64*. 10.1111/jpi.12455 29149454

[jev212410-bib-0022] Ma, J. , Chen, T. , Wu, S. , Yang, C. , Bai, M. , Shu, K. , Li, K. , Zhang, G. , Jin, Z. , He, F. , Hermjakob, H. , & Zhu, Y. (2019). iProX: An integrated proteome resource. Nucleic Acids Research, 47, D1211–D1217. 30252093 10.1093/nar/gky869 PMC6323926

[jev212410-bib-0023] Mills, E. L. , Kelly, B. , Logan, A. , Costa, A. S. H. , Varma, M. , Bryant, C. E. , Tourlomousis, P. , Däbritz, J. H. M. , Gottlieb, E. , Latorre, I. , Corr, S. C. , McManus, G. , Ryan, D. , Jacobs, H. T. , Szibor, M. , Xavier, R. J. , … O'Neill, L. A. (2016). Succinate dehydrogenase supports metabolic repurposing of mitochondria to drive inflammatory macrophages. Cell, 167, 457–45+. 10.1016/j.cell.2016.08.064 PMC5863951 27667687

[jev212410-bib-0024] Motwani, M. , Pesiridis, S. , & Fitzgerald, K. A. (2019). DNA sensing by the cGAS‐STING pathway in health and disease. Nature Reviews Genetics, 20, 657–674. 10.1038/s41576-019-0151-1 31358977

[jev212410-bib-0025] Nicolás‐Avila, J. A. , Lechuga‐Vieco, A. V. , Esteban‐Martínez, L. , Sánchez‐Díaz, M. , Díaz‐García, E. , Santiago, D. J. , Haas, S. , Wagman, A. , Arnelo, U. , Sutton, R. , Heuchel, R. L. , & Hidalgo, A. (2020). A network of macrophages supports mitochondrial homeostasis in the heart. Cell, 183, 94–9+. 10.1016/j.cell.2020.08.031 32937105

[jev212410-bib-0026] Norberg, K. J. , Nania, S. , Li, X. , Gao, H. , Szatmary, P. , Segersvard, R. , … Löhr, J. M. (2018). RCAN1 is a marker of oxidative stress. induced in acute pancreatitis. Pancreatology, 18, 734–741. 30139658 10.1016/j.pan.2018.08.005

[jev212410-bib-0027] Pagliuca, F. W. , Millman, J. R. , Gurtler, M. , Segel, M. , Van Dervort, A. , Ryu, J. H. , Peterson, Q. P. , Greiner, D. , & Melton, D. A. (2014). Generation of functional human pancreatic beta cells in vitro. Cell, 159, 428–439. 25303535 10.1016/j.cell.2014.09.040 PMC4617632

[jev212410-bib-0028] Pendharkar, S. A. , Mathew, J. , & Petrov, M. S. (2017). Age‐ and sex‐specific prevalence of diabetes associated with diseases of the exocrine pancreas: A population‐based study. Digestive and Liver Disease, 49, 540–544. 28110921 10.1016/j.dld.2016.12.010

[jev212410-bib-0029] Petrov, M. S. , & Yadav, D. (2019). Global epidemiology and holistic prevention of pancreatitis. Nature reviews Gastroenterology & hepatology, 16, 175–184. 30482911 10.1038/s41575-018-0087-5 PMC6597260

[jev212410-bib-0030] Sakaguchi, Y. , Inaba, M. , Kusafuka, K. , Okazaki, K. , & kehara, S. (2006). Establishment of animal models for three types of pancreatitis and analyses of regeneration mechanisms. Pancreas, 33, 371–381. 17079942 10.1097/01.mpa.0000236734.39241.99

[jev212410-bib-0031] Salvioli, S. , Ardizzoni, A. , Franceschi, C. , & Cossarizza, A. (1997). JC‐1, but not DiOC6(3) or rhodamine 123, is a reliable fluorescent probe to assess delta psi changes in intact cells: Implications for studies on mitochondrial functionality during apoptosis. Febs Letters, 411, 77–82. 9247146 10.1016/s0014-5793(97)00669-8

[jev212410-bib-0032] Sansone, P. , Savini, C. , Kurelac, I. , Chang, Q. , Amato, L. B. , Strillacci, A. , Stepanova, A. , Iommarini, L. , Mastroleo, C. , Daly, L. , Galkin, A. , Thakur, B. K. , Soplop, N. , Uryu, K. , Hoshino, A. , Norton, L. , … Bromberg, J. (2017). Packaging and transfer of mitochondrial DNA via exosomes regulate escape from dormancy in hormonal therapy‐resistant breast cancer. PNAS, 114, E9066–E9075. 29073103 10.1073/pnas.1704862114 PMC5664494

[jev212410-bib-0033] Santulli, G. (2018). Exosomal microRNA: The revolutionary endogenous Innerspace nanotechnology. Science Translational Medicine, 10, eaav9141. 30899414 10.1126/scitranslmed.aav9141 PMC6424121

[jev212410-bib-0034] Saravanan, P. B. , Vasu, S. , Yoshimatsu, G. , Darden, C. M. , Wang, X. , Gu, J. , Lawrence, M. C. , & Naziruddin, B. (2019). Differential expression and release of exosomal miRNAs by human islets under inflammatory and hypoxic stress. Diabetologia, 62, 1901–1914. 31372667 10.1007/s00125-019-4950-x

[jev212410-bib-0035] Stockwell, B. R. , Friedmann Angeli, J. P. , Bayir, H. , Bush, A. I. , Conrad, M. , Dixon, S. J. , Fulda, S. , Gascón, S. , Hatzios, S. K. , Kagan, V. E. , Noel, K. , Jiang, X. , Linkermann, A. , Murphy, M. E. , Overholtzer, M. , Oyagi, A. , … Zhang, D. D. (2017). Ferroptosis: A regulated cell death nexus linking metabolism. Redox Biology, and Disease. Cell, 171, 273–285. 28985560 10.1016/j.cell.2017.09.021 PMC5685180

[jev212410-bib-0036] Sun, L. , Wu, J. , Du, F. , Chen, X. , & Chen, Z. J. (2013). Cyclic GMP‐AMP synthase is a cytosolic DNA sensor that activates the type I interferon pathway. Science, 339, 786–791. 23258413 10.1126/science.1232458 PMC3863629

[jev212410-bib-0037] Tadokoro, T. , Ikeda, M. , Ide, T. , Deguchi, H. , Ikeda, S. , Okabe, K. , Ishikita, A. , Matsushima, S. , Koumura, T. , Yamada, K. I. , Imai, H. , & Tsutsui, H. (2020). Mitochondria‐dependent ferroptosis plays a pivotal role in doxorubicin cardiotoxicity. JCI Insight, *5*. 10.1172/jci.insight.132747 PMC7253028 32376803

[jev212410-bib-0038] Tadokoro, T. , Ikeda, M. , Ide, T. , Deguchi, H. , Ikeda, S. , Okabe, K. , Ishikita, A. , Matsushima, S. , Koumura, T. , Yamada, K. I. , Imai, H. , & Tsutsui, H. (2023). Mitochondria‐dependent ferroptosis plays a pivotal role in doxorubicin cardiotoxicity. JCI Insight, *8*. 10.1172/jci.insight.169756 PMC10070098 36946465

[jev212410-bib-0039] Tian, Y. , Gong, M. , Hu, Y. , Liu, H. , Zhang, W. , Zhang, M. , Hu, X. , Aubert, D. , Zhu, S. , Wu, L. , & Yan, X. (2020). Quality and efficiency assessment of six extracellular vesicle isolation methods by nano‐flow cytometry. Journal of Extracellular Vesicles, 9, 1697028. 31839906 10.1080/20013078.2019.1697028 PMC6896440

[jev212410-bib-0040] Tu, J. , Zhang, J. , Ke, L. , Yang, Y. , Yang, Q. , Lu, G. , Li, B. , Tong, Z. , Li, W. , & Li, J. (2017). Endocrine and exocrine pancreatic insufficiency after acute pancreatitis: Long‐term follow‐up study. BMC Gastroenterology [Electronic Resource], 17, 114. 29078749 10.1186/s12876-017-0663-0 PMC5658961

[jev212410-bib-0041] van der Vlist, M. , Raoof, R. , Willemen, H. , Prado, J. , Versteeg, S. , Martin Gil, C. , Vos, M. , Lokhorst, R. E. , Jeroen Pasterkamp, R. , Kojima, T. , Karasuyama, H. , Khoury‐Hanold, W. , Meyaard, L. , & Eijkelkamp, N. (2022). Macrophages transfer mitochondria to sensory neurons to resolve inflammatory pain. Neuron, 110, 613–626.e619. 34921782 10.1016/j.neuron.2021.11.020

[jev212410-bib-0042] Van Gassen, N. , Staels, W. , Van Overmeire, E. , De Groef, S. , Sojoodi, M. , Heremans, Y. , Leuckx, G. , Van de Casteele, M. , Van Ginderachter, J. A. , Heimberg, H. , & De Leu, N. (2015). Concise review: Macrophages: Versatile gatekeepers during pancreatic beta‐cell development, injury, and regeneration. Stem Cells Translational Medicin, 4, 555–563. 10.5966/sctm.2014-0272 PMC4449100 25848123

[jev212410-bib-0043] Wang, X. M. , Xu, F. , Kou, H. Y. , Zheng, Y. W. , Yang, J. , Xu, Z. , Fang, Y. , Sun, W. , Zhu, S. , Jiang, Q. , Wei, X. , & Xu, Y. (2023). Stromal cell‐derived small extracellular vesicles enhance radioresistance of prostate cancer cells via interleukin‐8‐induced autophagy. Journal of Extracellular Vesicles, 12, e12342. 37387557 10.1002/jev2.12342 PMC10312072

[jev212410-bib-0044] Wu, J. , Zhang, L. , Shi, J. , He, R. , Yang, W. , Habtezion, A. , Niu, N. , Lu, P. , & Xue, J. (2020). Macrophage phenotypic switch orchestrates the inflammation and repair/regeneration following acute pancreatitis injury. EBioMedicine, 58, 102920. 32739869 10.1016/j.ebiom.2020.102920 PMC7399125

[jev212410-bib-0045] Xie, Y. , Yu, L. , Cheng, Z. , Peng, Y. , Cao, Z. , Chen, B. , Duan, Y. , & Wang, Y. (2022). SHED‐derived exosomes promote LPS‐induced wound healing with less itching by stimulating macrophage autophagy. Journal of Nanobiotechnology, 20, 239. 35597946 10.1186/s12951-022-01446-1 PMC9124392

[jev212410-bib-0046] You, D. G. , Lim, G. T. , Kwon, S. , Um, W. , Oh, B. H. , Song, S. H. , Lee, J. , Jo, D. G. , Cho, Y. W. , & Park, J. H. (2021). Metabolically engineered stem cell‐derived exosomes to regulate macrophage heterogeneity in rheumatoid arthritis. Science Advances, 7, eabe0083. 34078596 10.1126/sciadv.abe0083 PMC8172131

[jev212410-bib-0047] Yuan, D. F. , Zhao, Y. L. , Banks, W. A. , Bullock, K. M. , Haney, M. , Batrakova, E. , & Kabanov, A. V. (2017). Macrophage exosomes as natural nanocarriers for protein delivery to inflamed brain. Biomaterials, 142, 1–12. 28715655 10.1016/j.biomaterials.2017.07.011 PMC5603188

[jev212410-bib-0048] Zecchini, V. , Paupe, V. , Herranz‐Montoya, I. , Janssen, J. , Wortel, I. M. N. , Morris, J. L. , Ferguson, A. , Roy Chowdury, S. , Segarra‐Mondejar, M. , Costa, A. S. H. , Pereira, G. C. , Tronci, L. , Young, T. , Nikitopoulou, E. , Yang, M. , Bihary, D. , Caicci, F. , Nagashima, S. , Speed, A. , … Frezza, C. (2023). Fumarate induces vesicular release of mtDNA to drive innate immunity. Nature, 615, 499–506. 36890229 10.1038/s41586-023-05770-w PMC10017517

[jev212410-bib-0049] Zhang, R. , Kang, R. , & Tang, D. (2021). The STING1 network regulates autophagy and cell death. Signal Transduction and Targeted Therapy, 6, 208. 34078874 10.1038/s41392-021-00613-4 PMC8172903

