# Correction to “Transfer of inflammatory mitochondria via extracellular vesicles from M1 macrophages induces ferroptosis of pancreatic beta cells in acute pancreatitis”

**DOI:** 10.1002/jev2.12441

**Published:** 2024-04-22

Gao Y., Mi N., Wu W., Zhao Y., Fan F., Liao W., Ming Y., Guan W., & Bai C. (2024). Transfer of inflammatory mitochondria via extracellular vesicles from M1 macrophages induces ferroptosis of pancreatic beta cells in acute pancreatitis. Journal of Extracellular Vesicles, 13, e12410. https://doi.org/10.1002/jev2.12410

In the originally published article, there was an error in the address of author Weijun Guan. The correct address is as follows:

Weijun Guan

Institute of Animal Sciences, Chinese Academy of Agricultural Sciences

Yuanmingyuan West Road, Haidian District

Beijing 100193, R.P. China

Email: guanweijun@caas.cn

This information has been updated in the online version of the article.

We apologize for this error.

