# Supplementary material for: Transfer of inflammatory mitochondria via extracellular vesicles from M1 macrophages induces ferroptosis of pancreatic beta cells in acute pancreatitis
Source: J Extracell Vesicles. 2024 Feb 6;13(2):e12410. doi: 10.1002/jev2.12410 (PMC10847061; doi:10.1002/jev2.12410)
Supplement: Supplementary file 1 — Supplementary Information [file JEV2-13-e12410-s002.pdf]

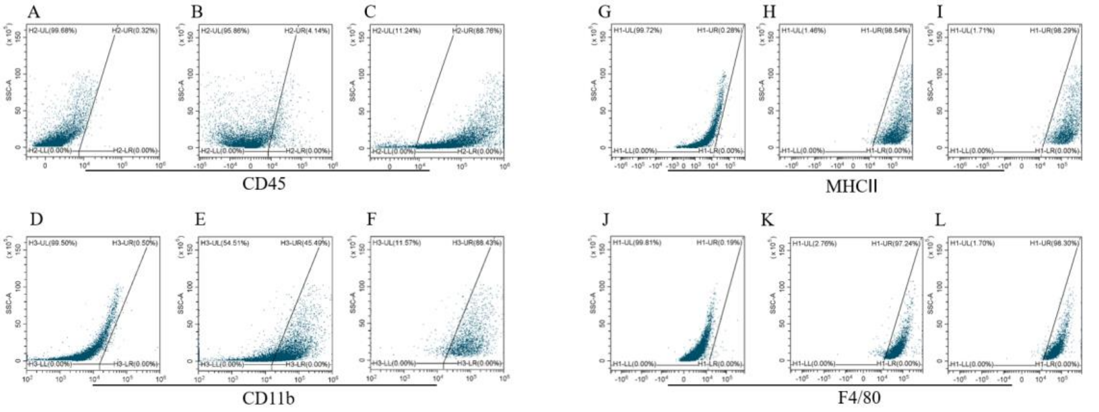

Figure S1. Example of the gating strategy to sort pancreatic resident macrophages.

A. Negative control for CD45 antibody. B. FCM analysis for CD45-positive cells in single-cell suspension derived from pancreatic tissue. C. The CD45-positive subpopulation from pancreatic tissue was isolated using immunomagnetic beads, and subsequently subjected to FCM analysis. D. Negative control for CD11b antibody. E. FCM analysis for CD11b-positive cells in CD45-positive subpopulation. C. The CD11b-positive subpopulation from CD45-positive subpopulation was isolated using immunomagnetic beads, and subsequently subjected to FCM analysis. G. Negative control for MHCⅡ antibody. H. FCM analysis for MHCⅡ-positive cells in CD11b-positive subpopulation. I. The MHCⅡ-positive subpopulation from CD11b-positive subpopulation was isolated using immunomagnetic beads, and subsequently subjected to FCM analysis. J. Negative control for F4/80 antibody. K. FCM analysis for F4/80-positive cells in MHCⅡ-positive subpopulation. I. The F4/80-positive subpopulation from MHCⅡ-positive subpopulation was isolated using immunomagnetic beads, and subsequently subjected to FCM analysis.

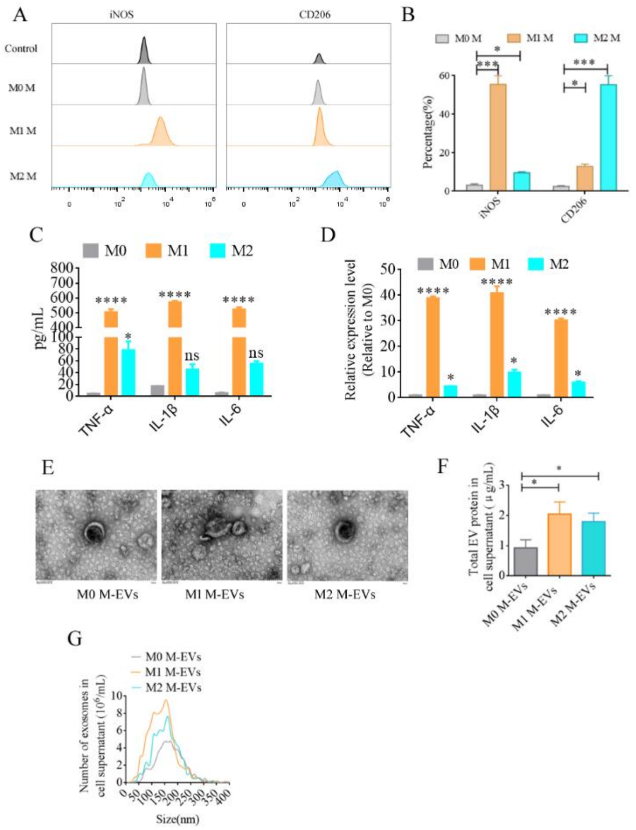

Figure S2 The polarization of macrophages and biological characteristics of EVs from polarized macrophages.

A and B. Flow cytometry of the M1 (iNOS) and M2 (CD206) macrophage markers in macrophages (left), quantification of the expression levels of iNOS and CD206 in polarized macrophages (n=3). C. Electron microscopy images of EVs from polarized macrophages. C. EVs protein concentrations of EVs from polarized macrophages. D. Size distributions of EVs from polarized macrophages. n=3. *$P < 0.05$, **$P < 0.01$, ***$P < 0.001$, ****$P < 0.0001$. ns, not significant.

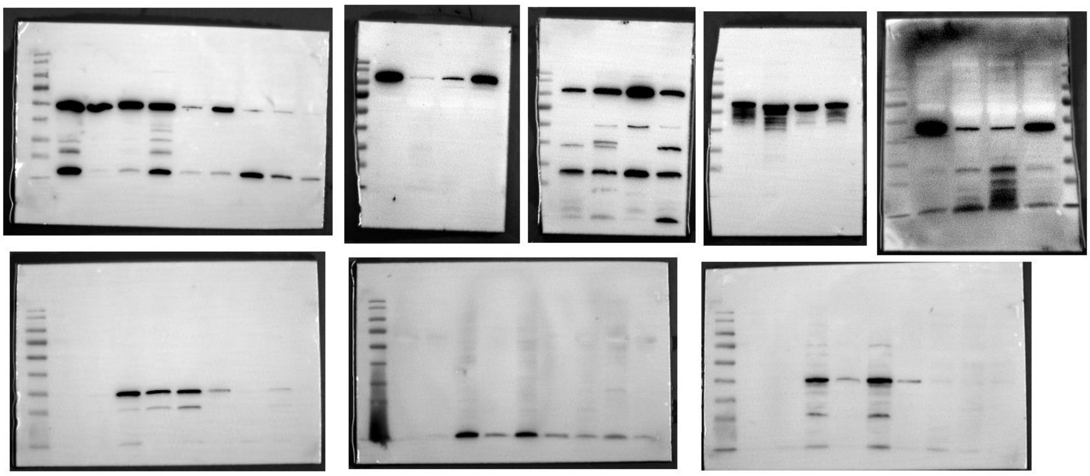

Figure S3. The complete blot images corresponding to Figure 1 B and D.

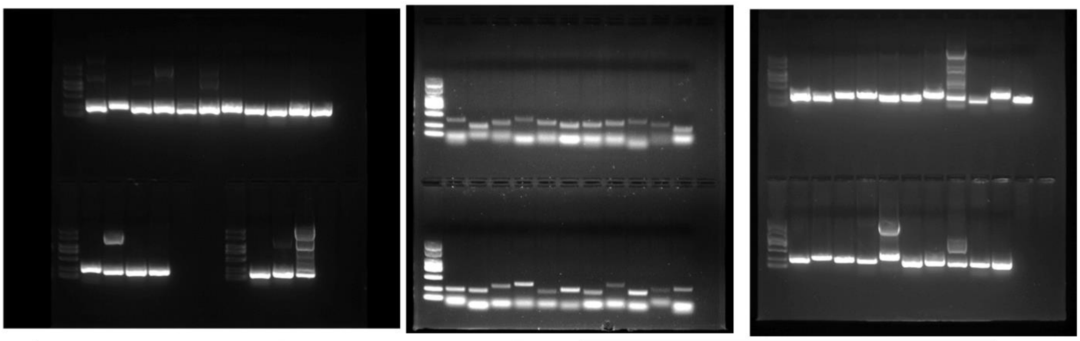

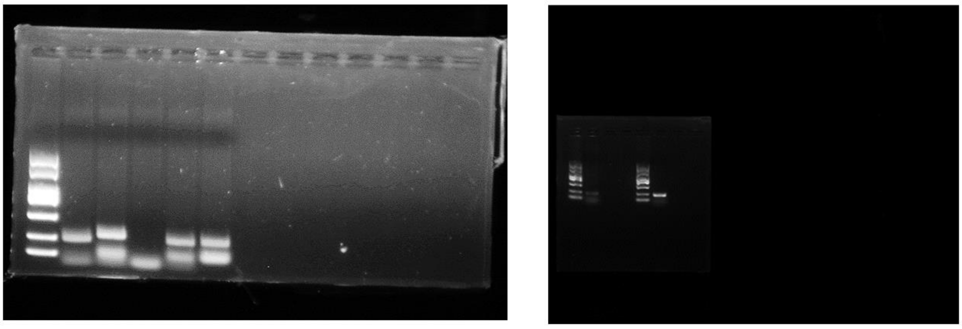

Figure S4. The complete gel electrophoresis image corresponding to Figure 1 E.

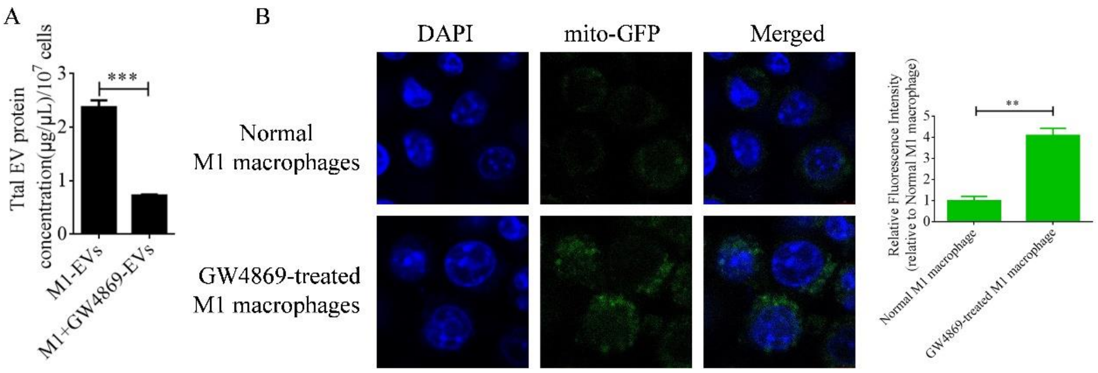

Figure S5. GW4869 inhibits extracellular vesicle release in M1 macrophages. A. The concentration of EV proteins indicates that GW4869 effectively inhibits the release of EVs from M1 macrophages. B. Changes in GFP fluorescence intensity in M1 macrophages before and after treatment with GW4869 mito-GFP fluorescence intensity. These results suggested that mitochondrial transfer occurred through the release of EVs. ***P* < 0.01, ****P* < 0.01.

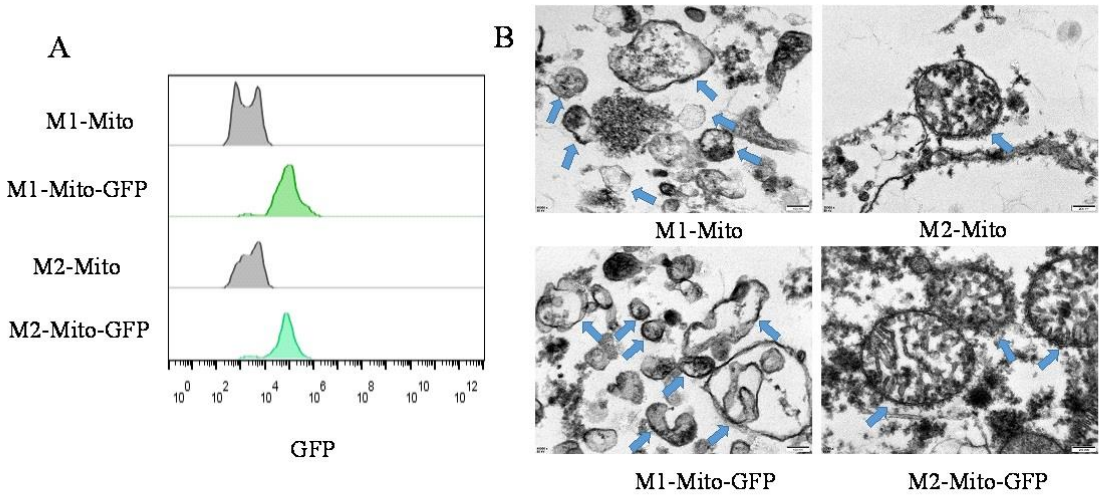

Figure S6. Biological characteristics of secreted mitochondria from macrophages. A. mitochondrial GFP detected by FCM. B. Analyzing the morphological characteristics of secreted mitochondria derived from macrophages by transmission electron microscopy.

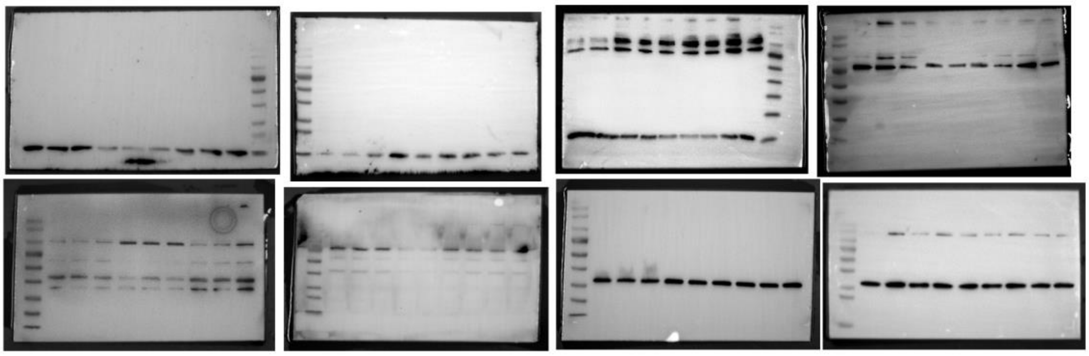

Figure S7. The complete blot images corresponding to Figure 7D.

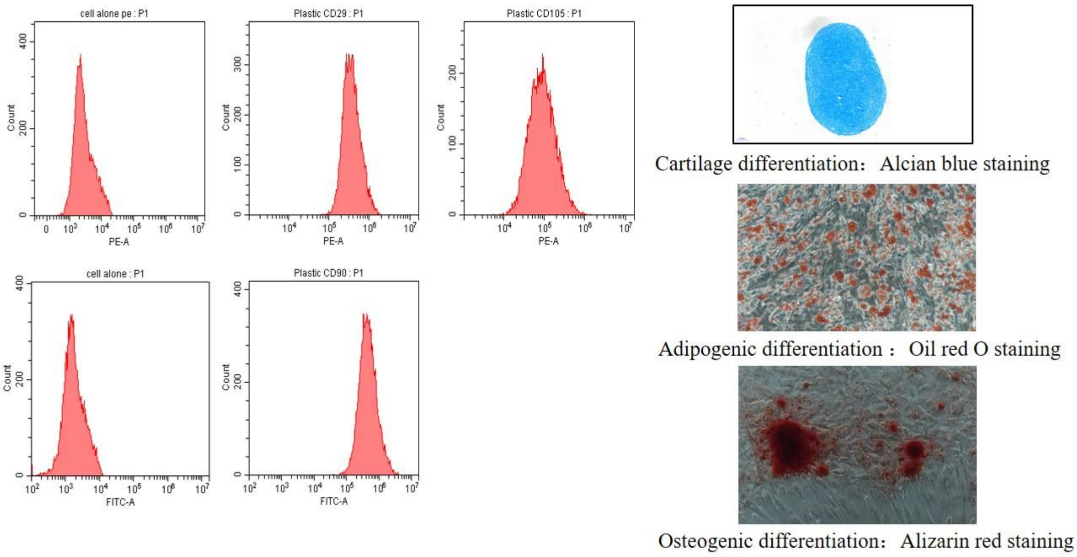

Figure S8. The analysis of biological characteristics in human ADSCs.

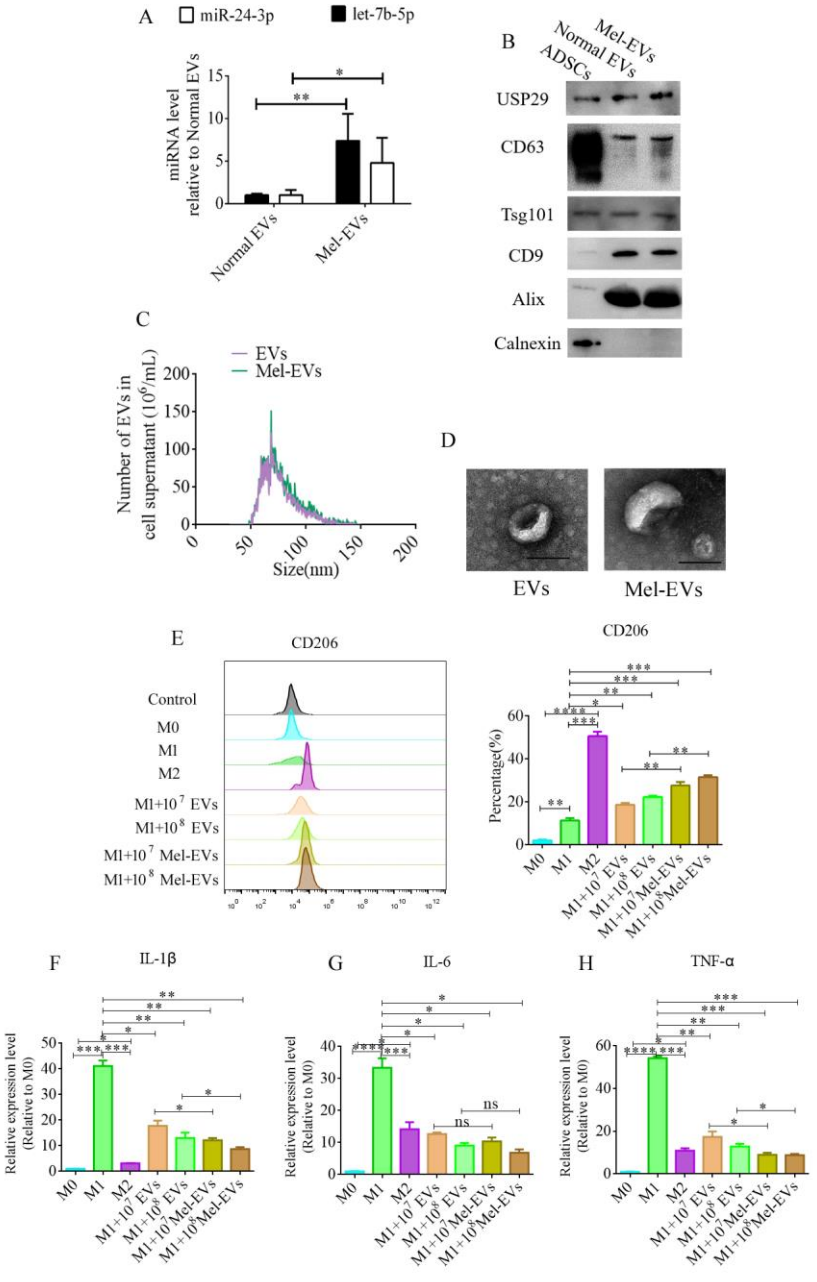

Figure S9. The analysis of biological characteristics in EVs derived from human ADSCs before and after treatment by melatonin. A. the level of special miRNAs (let-7b-5p and miR-24-3p) in EVs derived from human ADSCs before and after treatment by melatonin. B. The protein level of protease (USP29) and EV markers in EVs derived from human ADSCs before and after treatment by melatonin. C. Size distributions of EVs derived from human ADSCs before and after treatment by melatonin. D. Representative electron microscopy images of EVs derived from human ADSCs before and after treatment by melatonin. E. Flow cytometry of the M2

(CD206) macrophage markers in PRMs after treatment with EVs derived from human ADSCs before and after treatment by melatonin. F, G and H. n=3. **P* < 0.05, ***P* < 0.01, ****P* < 0.001, *****P* < 0.0001. ns, not significant.

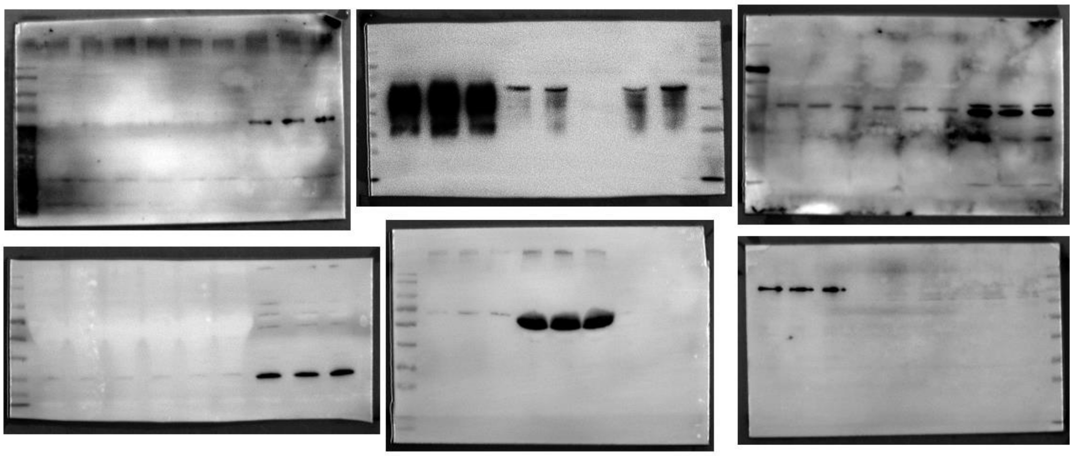

Figure S10. The complete blot images corresponding to Figure S 9B.

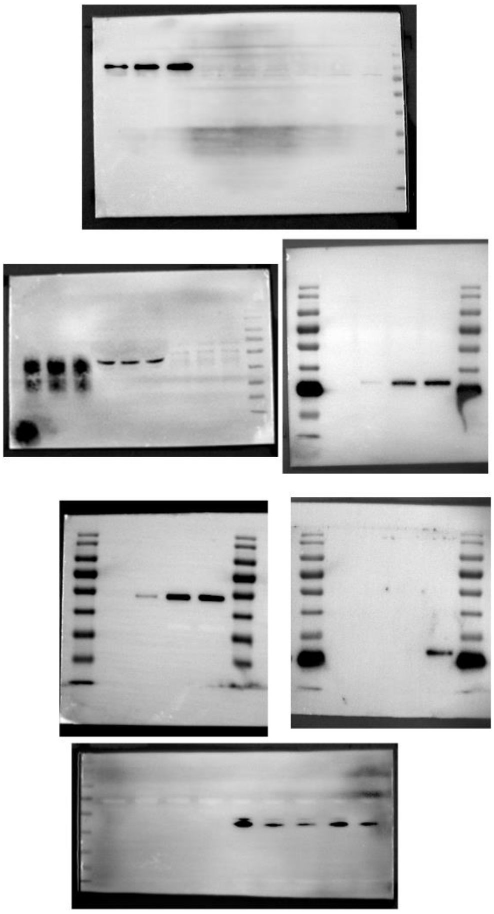

Figure S11. The complete blot images corresponding to Figure 8B.

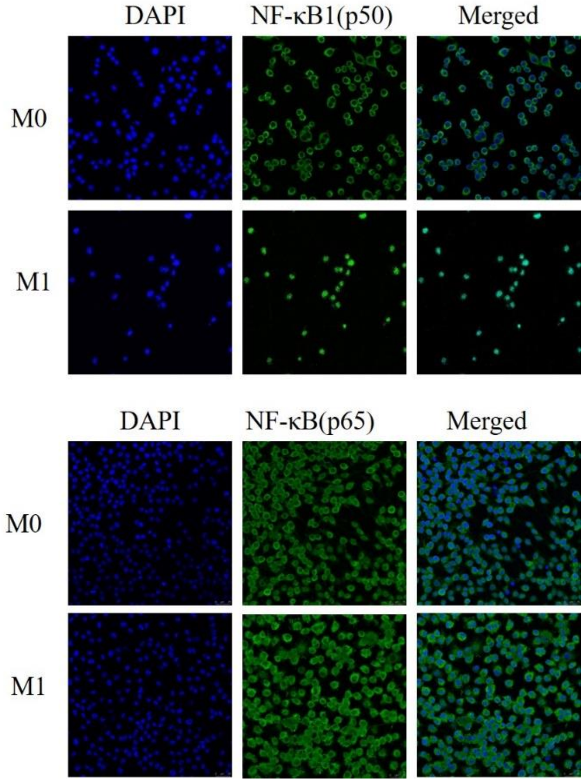

Figure S12. The immunofluorescence analysis of NF-κB (p65) and NF-κB (p50) were detected in Macrophages after M1 polarization.

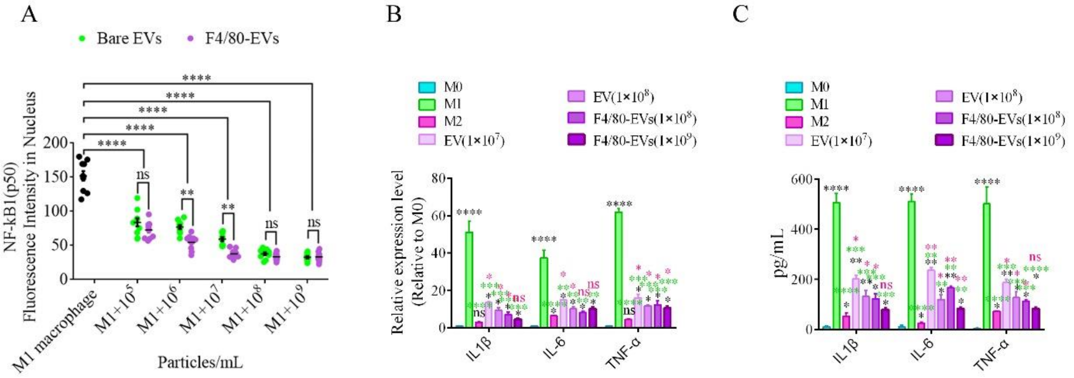

Figure S13. IL-1β, TNF-α, and IL-6 were detected with real time PCR and ELISA in M1 macrophages and cell suspension.

A. Analysis for fluorescence intensity of NF-κB1(p50) in the nucleus from M1 macrophages after different treatments (n=10). B.IL-1β, TNF-α, and IL-6 were detected with real time PCR in M1 macrophages after different treatment. C. IL-1β, TNF-α, and IL-6 were detected with ELISA in cell suspension of M1 macrophages after different treatment. Black superscript, M0 vs. each group; Laurel green

superscript, M1 vs. each group; Purple superscript, M2-EVs vs. each group. n=3. **P* < 0.05, ***P* < 0.01, ****P* < 0.001, *****P* < 0.0001. ns, not significant.

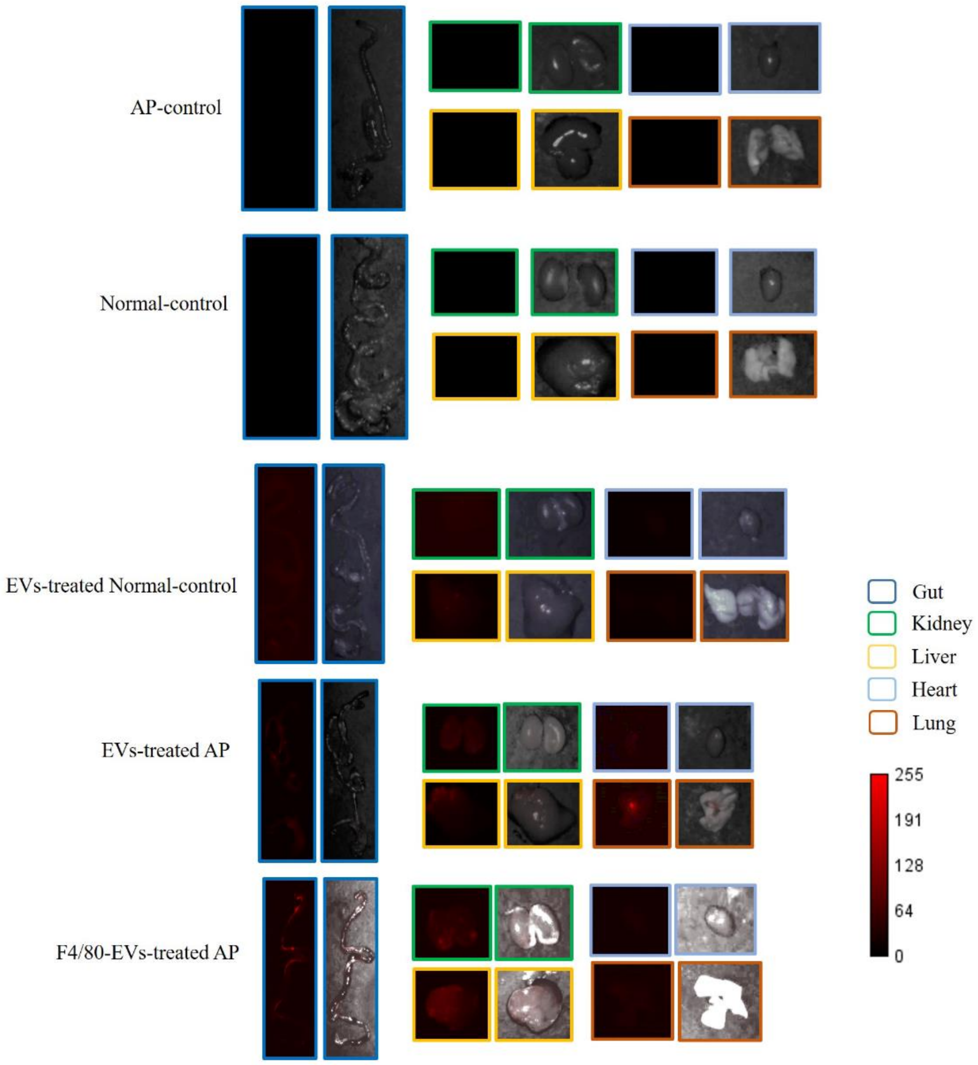

Figure S14. The organs fluorescence imaging of EVs and F4/80-EVs (1×10^9 particles per head) in AP mice or normal mice.

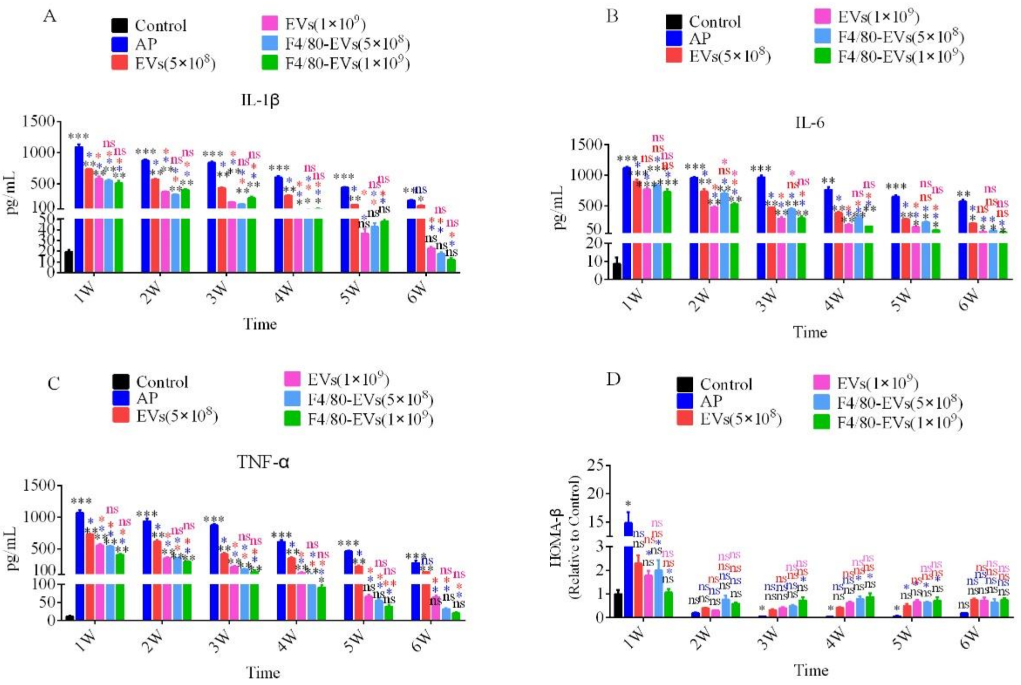

Figure S15. The detection of inflammatory factors in plasma and the analysis of the functioning of β cells in AP mice after different treatment.

A, B and C. the inflammatory factor, IL-1β, IL-6 and TNF-α, were detected with ELISA in plasma of AP mice after different treatment. D. the homeostasis model assessment (HOMA) was used to evaluate the function of β cell in AP mice after different treatment. Black superscript, Control vs. each group; Blue superscript, AP vs. each group; Red superscript, EVs(5×10^8) vs. each group. Pink superscript, EVs(1×10^9) vs. each group. n=3, *$P < 0.05$, **$P < 0.01$, ***$P < 0.001$, ****$P < 0.0001$. ns, not significant.
