# Supplementary material for: Transfer of inflammatory mitochondria via extracellular vesicles from M1 macrophages induces ferroptosis of pancreatic beta cells in acute pancreatitis
Source: J Extracell Vesicles. 2024 Feb 6;13(2):e12410. doi: 10.1002/jev2.12410 (PMC10847061; doi:10.1002/jev2.12410)
Supplement: Supplementary file 2 — Supplementary Information [file JEV2-13-e12410-s001.docx]

Table S2 presents the primer list for the amplification of the entire mitochondrial DNA

| Primer | Sequence (5’→3’) | Product length |
| --- | --- | --- |
| mt1-F | AACACAAAGGTTTGGTCCTGGC | 251 |
| mt1-R | TGGCTGGCACGAAATTTACCAACC | 251 |
| mt2-F | CCCCGCTCTACCTCACCATCTC | 267 |
| mt2-R | CGGTGTGTGCGTACTTCATTGC | 267 |
| mt3-F | ACTAATCCTAGCCCTAGCCCTACAC | 252 |
| mt3-R | TCGTTTGGTTTCGGGGTTTCTAGC | 252 |
| mt4-F | GCAGCCACCAATAAAGAAAGCGTTC | 273 |
| mt4-R | TTCCTTTAGGCATTCCGGTGTTGG | 273 |
| mt5-F | GAACGGCTAAACGAGGGTCCAAC | 206 |
| mt5-R | TTTTATTCTCCGAGGTCACCCCAAC | 206 |
| mt6-F | CGGAGCAATCCAGGTCGGTTTC | 289 |
| mt6-R | GGAAGGCTATGGCGATTAGAATGGG | 289 |
| mt7-F | TGAGTTCCCCTACCAATACCACACC | 285 |
| mt7-R | GGGTCAGGCTGGCAGAAGTAATC | 285 |
| mt8-F | acattcctatggatccgagcatct | 223 |
| mt8-R | tcctagaaataagagggcttgaacct | 223 |
| mt9-F | ACCCCGAAAACGTTGGTTTAAATCC | 237 |
| mt9-R | TGAGGCTGTTGCTTGTGTGACG | 237 |
| mt10-F | GGGCATGAGGAGGACTTAACCAAAC | 289 |
| mt10-R | AAGAATCCTGTTAGTGGTGGAAGGC | 289 |
| mt11-F | AGCACAATAACCCTACCCCTAGCC | 264 |
| mt11-R | TTCGGCGGTAGAAGTAGATTGAAGC | 264 |
| mt12-F | TTCGGAGCCTGAGCGGGAATAG | 329 |
| mt12-R | GGTGGGTAGACTGTTCATCCTGTTC | 329 |
| mt13-F | ctttctttgatcccgctggagg | 222 |
| mt13-R | tgtggtgggctcatacaataaagc | 222 |
| mt14-F | ACAGTTGGTGGTCTAACCGGAATTG | 294 |
| mt14-R | AGCATCTGGGTAGTCTGAGTAGCG | 294 |
| mt15-F | GCCCTCCACCATATCACACATTCG | 221 |
| mt15-R | GTAGACCAAGTTGGAATGGGTAGGC | 221 |
| mt16-F | gggcaccaatgatactgaagc | 241 |
| mt16-R | gatttagtcggcctgggatg | 241 |
| mt17-F | AAACATTCCCACTGGCACCTTCAC | 268 |
| mt17-R | TCGTCCTTTTGGTGTGTGGATTAGC | 268 |
| mt18-F | ACATGATCTAGGAGGCTGCTGACC | 214 |
| mt18-R | TTCTGAAGCTTGGAGGATGGTGAAG | 214 |
| mt19-F | TGAAGCCGCAGCATGATACTGAC | 306 |
| mt19-R | ATTGAGAATGGTAGACGTGCAGAGC | 306 |
| mt20-F | TGCCATCTACCTTCTTCAACCTCAC | 203 |
| mt20-R | GGCTGCGAAAACTAAGATGGTGATG | 203 |
| mt21-F | agcctaacacttctatgacaaaccg | 278 |
| mt21-R | agtgttgggattaaggttgcttca | 278 |
| mt22-F | TCACAACACACACCTTAGACGCTTC | 349 |
| mt22-R | ACAAGTGCTATGTGGCTAACTGAGG | 349 |
| mt23-F | GCCCGAGGACTTCAAATGGTCTTC | 268 |
| mt23-R | GTTCTCGTGTGTGTGAGGGTTGG | 268 |
| mt24-F | atccattggtcttaggaaccaaaaacc | 270 |
| mt24-R | tgaatttatggtgactcagtgccagg | 270 |
| mt25-F | ATTGGATGATGGTACGGACGAACAG | 385 |
| mt25-R | GGGCTCCGAGGCAAAGTATAGTTG | 385 |
| mt26-F | CCAACCACACCTAGCATTCCTACAC | 271 |
| mt26-R | TAGTAGGGCTCAGGCGTTGGTG | 271 |
| mt27-F | attccaccaaccagcattcc | 205 |
| mt27-R | ttatgggtgtaatgcggtgaa | 205 |
| mt28-F | CCCCAATCCCTCCTTCCAACATAAC | 387 |
| mt28-R | TTTTGGTTGGTTGTCTTGGGTTAGC | 387 |
| mt29-F | CCTACCTGCCCCATCCAACATTTC | 243 |
| mt29-R | CCTCGTCCGACATGAAGGAATAAGC | 243 |
| mt30-F | ATCGTTCACCTCCTCTTCCTCCAC | 275 |
| mt30-R | GGGATTGAGCGTAGAATGGCGTATG | 275 |
| mt31-F | AGCCTAATATTCCGCCCAATCACAC | 307 |
| mt31-R | GGTGCTGGTGGTGGGGAGTAG | 307 |
| mt32-F | CCAACAACCCGCCCACCAATG | 252 |
| mt32-R | GCCTTGACGGCTATGTTGATGAAAG | 252 |
| mt33-F | agacgcacctacggtgaaga | 208 |
| mt33-R | gaattgatcaggacatagggtttg | 208 |
| mt34-F | AGACACCTTGCCTAGCCACACC | 396 |
| mt34-R | CCGCCAAGTCCTTTGAGTTTTAAGC | 396 |
| mt35-F | TTGAATTGAGCAATGAAGTACGCACAC | 240 |
| mt35-R | TGTAGGGCTAGGGCTAGGATTAGTTC | 240 |
| mt36-F | AGAATTACAGCTAGAAACCCCGAAACC | 321 |
| mt36-R | TTTCTTTATTGGTGGCTGCTTTTAGGC | 321 |
| mt37-F | ACACCGGAATGCCTAAAGGAAAGATC | 221 |
| mt37-R | GGACCCTCGTTTAGCCGTTCATG | 221 |
| mt38-F | agggataacagcgcaatcct | 202 |
| mt38-R | cctttcgtactgggagaaatcg | 202 |
| mt39-F | ACATTGTTGGTCCATACGGCATTTTAC | 267 |
| mt39-R | ATTTGGAGTTTGAGGCTCATCCTGATC | 267 |
| mt40-F | TCTGCCAGCCTGACCCATAGC | 325 |
| mt40-R | ATCGTAACGGAAGCGTGGATAAGATG | 325 |
| mt41-F | CTCGCCCCATTCCACTTCTGATTAC | 200 |
| mt41-R | TGTGTTTGGTTAAGTCCTCCTCATGC | 200 |
| mt42-F | GGCCTTCCACCACTAACAGGATTC | 266 |
| mt42-R | GGGGCTAGGGGTAGGGTTATTGTG | 266 |
| mt43-F | ACCTCAACTAGATTGGCAGGAA | 337 |
| mt43-R | TACCCACTATTCCCGCTCAGG | 337 |
| mt44-F | ATGAACAGTCTACCCACCTCTAGCC | 283 |
| mt44-R | GAAAGTTGTGTTTAGGTTGCGGTCTG | 283 |
| mt45-F | GAGCCCACCACATATTCACAGTAGG | 239 |
| mt45-R | AAGCACGATGTCAAGGGATGAGTTG | 239 |
| mt46-F | CCACTTCGCCATCATATTCGTAGGAG | 264 |
| mt46-R | GGAGGGCAGCCATGAAGTCATTC | 264 |
| mt47-F | GCTCACTTGCCCACTTCCTTCC | 287 |
| mt47-R | TACTAGGAGGGTGAATACGTAGGCTTG | 287 |
| mt48-F | AATGCGGATTCGACCCTACAAGC | 333 |
| mt48-R | GGTGAGGTTGAAGAAGGTAGATGGC | 333 |
| mt49-F | CCAACTCCATAAGCTCCATACCAATCC | 276 |
| mt49-R | TCGTCGGTTTGTCATAGAAGTGTTAGG | 276 |
| mt50-F | TCGGTTCTATTCCACTGCTAATTGCC | 202 |
| mt50-R | TCAACATGGGCTTTTGGTAGTCATAGG | 202 |
| mt51-F | CACTAATCGCCTACTCCTCAGTTAGC | 226 |
| mt51-R | CATCATGTGGCTATAAGTGGGAAGACC | 226 |
| mt52-F | AATTAACCTCCAACCCTCACACACAC | 215 |
| mt52-R | ATGGAAGCATGAATTAGCAGTTCTTGC | 215 |
| mt53-F | AATTACAACCTGGCACTGAGTCACC | 305 |
| mt53-R | CGTCTGTTCGTCCGTACCATCATC | 305 |
| mt54-F | CCTACACCAGTTTCAGCACTACTACAC | 341 |
| mt54-R | TCGTCTGCCAGGCTATGAATGATTG | 341 |
| mt55-F | GTGAAGGCTTTAATGCTAACCCAAGAC | 216 |
| mt55-R | GGCAGGTAGGTCAATGAATGAGTGG | 216 |
| mt56-F | TCGCAGTCATAGCCACAGCATTTATAG | 239 |
| mt56-R | GAGGAAGAGGAGGTGAACGATTGC | 239 |
| mt57-F | ACTAGGAGACCCAGACAACTACATACC | 207 |
| mt57-R | CGGAATATTAGGCTTCGTTGCTTTGAG | 207 |
| mt58-F | AAGAAGGAGCTACTCCCCACCAC | 353 |
| mt58-R | TTGTTGGTTTCACGGAGGATGGTAG | 353 |
| mt59-F | TCAGGGCCATCAAATGCGTTATCG | 219 |
| mt59-R | GGGTTTTGCGGACTAATGATTCTTCAC | 219 |
| mt60-F | CCCCTCCTCTTAATGCCAAACCC | 286 |
| mt60-R | GCCAGGACCAAACCTTTGTGTTTATG | 286 |
| mt61-F | CGAGCATCTTATCCACGCTTCCG | 305 |
| mt61-R | GGCCCGATAGCTTAATTAGCTGAC | 305 |
| mt62-F | CATAATTGCTCTCCCCTCTCTACGC | 214 |
| mt62-R | TGGCAGAACGACTCGGTTATCAAC | 214 |
| mt63-F | AAACATTCCCACTGGCACCTTCAC | 268 |
| mt63-R | TCGTCCTTTTGGTGTGTGGATTAGC | 268 |
| mt64-F | tcaccatcctccaagcttca | 208 |
| mt64-R | cagtatcatgctgcggcttc | 208 |
| mt65-F | CCAACGCCTGAGCCCTACTAATTAC | 235 |
| mt65-R | ATTGTGAGGACTGGAATGCTGGTTG | 235 |
| mt66-F | ACCATCCCAAAATCCACCTCAACTC | 247 |
| mt66-R | TTGGAAGGAGGGATTGGGGTAGC | 247 |
| IL-1β-F | CTCGCAGCAGCACATCAACAAG | 94 |
| IL-1β-R | CCACGGGAAAGACACAGGTAGC | 94 |
| IL-10-F | GGTTGCCAAGCCTTATCGGAAATG | 133 |
| IL-10-R | GCCGCATCCTGAGGGTCTTC | 133 |
| TNF-α-F | ACGCTCTTCTGTCTACTGAACTTCG | 113 |
| TNF-α-R | TGGTTTGTGAGTGTGAGGGTCTG | 113 |
